# Variation in global COVID-19 symptoms by geography and by chronic disease: A global survey using the COVID-19 Symptom Mapper

**DOI:** 10.1016/j.eclinm.2022.101317

**Published:** 2022-03-06

**Authors:** Balasundaram Kadirvelu, Gabriel Burcea, Jennifer K. Quint, Ceire E. Costelloe, A. Aldo Faisal

**Affiliations:** a Brain & Behaviour Lab, Dept. Of Computing, Imperial College London, London, UK; b Global Digital Health Unit, School of Public Health, Imperial College London, London, UK; c National Heart and Lung Institute, Imperial College London, London, UK; d Brain & Behaviour Lab, Dept. Of Computing & Dept. Of Bioengineering, UKRI Centre for Doctoral Training in AI for Healthcare, and the Global Covid Observatory, Imperial College London, UK and MRC London Institute for Medical Sciences, London, UK; e Institute for Artificial & Human Intelligence, University of Bayreuth, Bayreuth, Germany

**Keywords:** COVID-19, COVID-19 symptoms, COVID symptoms mapper, COVID symptoms survey, COVID symptom profile, Comorbidities, COVID-19, The Coronavirus Disease that first appeared in 2019 caused by the SARS-CoV-2 coronavirus., WHO, World Health Organization, a specialized agency of the United Nations responsible for international public health., PCR, **Polymerase chain reaction**

## Abstract

**Background:**

COVID-19 is typically characterised by a triad of symptoms: cough, fever and loss of taste and smell, however, this varies globally. This study examines variations in COVID-19 symptom profiles based on underlying chronic disease and geographical location.

**Methods:**

Using a global online symptom survey of 78,299 responders in 190 countries between 09/04/2020 and 22/09/2020, we conducted an exploratory study to examine symptom profiles associated with a positive COVID-19 test result by country and underlying chronic disease (single, co- or multi-morbidities) using statistical and machine learning methods.

**Findings:**

From the results of 7980 COVID-19 tested positive responders, we find that symptom patterns differ by country. For example, India reported a lower proportion of headache (22.8% vs 47.8%, p<1e-13) and itchy eyes (7.3% vs. 16.5%, p=2e-8) than other countries. As with geographic location, we find people differed in their reported symptoms if they suffered from specific chronic diseases. For example, COVID-19 positive responders with asthma (25.3% vs. 13.7%, p=7e-6) were more likely to report shortness of breath compared to those with no underlying chronic disease.

**Interpretation:**

We have identified variation in COVID-19 symptom profiles depending on geographic location and underlying chronic disease. Failure to reflect this symptom variation in public health messaging may contribute to asymptomatic COVID-19 spread and put patients with chronic diseases at a greater risk of infection. Future work should focus on symptom profile variation in the emerging variants of the SARS-CoV-2 virus. This is crucial to speed up clinical diagnosis, predict prognostic outcomes and target treatment.

**Funding:**

We acknowledge funding to AAF by a UKRI Turing AI Fellowship and to CEC by a personal NIHR Career Development Fellowship (grant number NIHR-2016-090-015). JKQ has received grants from The Health Foundation, MRC, GSK, Bayer, BI, Asthma UK-British Lung Foundation, IQVIA, Chiesi AZ, and Insmed. This work is supported by BREATHE - The Health Data Research Hub for Respiratory Health [MC_PC_19004]. BREATHE is funded through the UK Research and Innovation Industrial Strategy Challenge Fund and delivered through Health Data Research UK. Imperial College London is grateful for the support from the Northwest London NIHR Applied Research Collaboration. The views expressed in this publication are those of the authors and not necessarily those of the NIHR or the Department of Health and Social Care.

**Research in context**

**Evidence before this study**

We searched the literature on COVID-19 symptom mappers using the terms “covid symptoms” and “covid symptom profile” from 1^st^ January 2020 to 15^th^ December 2020. A Cochrane review found data on 84 signs and symptoms in 44 studies. An early meta-analysis of epidemiological variation in COVID-19 inside and outside China found that important symptom differences existed in patients in China compared to other countries and recommended that clinical symptoms of COVID-19 should not be generalized to fever, shortness of breath and cough only. This is the first study to explore symptoms among those who test positive for COVID-19 by geolocation and underlying chronic disease.

**Added value of this study**

We find that across countries and based on underlying chronic diseases, there are differences in symptom profiles at presentation, that cannot be fully explained by the different chronic disease profiles of these countries.

**Implications of all the available evidence**

As SARS-CoV-2 mutates, and new variants emerge in the global population it is essential to understand the symptom profile in different populations. The simple triad of COVID symptoms may contribute to a false sense of security and therefore surveys, such as reported here, are key to rapidly understand how symptomatology is changing.

Alt-text: Unlabelled box

## Introduction

Since the start of the COVID-19 pandemic, most testing has been triggered by a classical triad of symptoms, which were first observed in COVID-19 patients who were hospitalised. However, grouping patients based on clinical characteristics is crucial for clinical practice, to allow selection of diagnostic tests and to predict prognosis. Web-based symptom checkers have become popular in the context of the novel COVID-19 pandemic, as access to physicians is reduced, concern in the population is high, and large amounts of misinformation are circulating social media.^1^ On COVID-19 symptom checker web pages, users are asked a series of COVID-19–specific questions; upon completion, an association between the answers and COVID-19 is given alongside in some instances, behavioural recommendations, such as self-isolation. In this context, COVID-19 symptom checkers can be valuable tools for pre-assessment and screening.

However, with COVID-19, important biological differences are likely to exist between patient subgroups, as is seen in other forms of critical illness.^2^^,^^3^ This has been demonstrated where highly significant subgroup effects were observed in the first drug trial to demonstrate an improvement in mortality, dexamethasone.^4^ People in different locations/cultures may perceive symptoms differently, and underlying diseases may mask/alter certain “typical” covid symptoms. Additionally, subgroups of the population are at higher risk of both developing COVID-19 and experiencing more severe infection, people aged 60 years and older; those living in long-term care facilities; and people with underlying health conditions, such as hypertension, diabetes, cardiovascular disease, chronic respiratory disease and weakened immune systems.^5^ On a global level, as new variants of COVID-19 arise in different parts of the world, it is hypothesised that symptom profiles may differ according to variant, and indeed geographical location.^6^

It is important to characterise the symptoms of severe COVID-19 and to identify clinical subgroups. This will speed up diagnosis, enable more precise prediction of outcomes, and target treatment. The aim of this study was to examine clustering of COVID-19 symptoms based on underlying chronic disease and geographical location.

## Methods

### Study setting and participants

The company Your.MD launched a web-based COVID-19 Symptom Mapper ^7^ survey in partnership with Imperial College London's Global Covid Observatory during the peak of the global pandemic in April 2020 to better understand how the new disease is affecting communities worldwide. An online (web-based) questionnaire was carried out. To minimise selection bias and improve the generalisability of the results, the following strategies were employed: The questions were asked in lay-person language, so they were easy to understand, and the questionnaire was translated into six major languages, namely, Hindi, Arabic, Spanish, Portuguese, French and Urdu. The Your.MD Covid-19 symptom mapper followed guidelines to the design and application of online questionnaire surveys suggested in the literature.^8^^,^^9^ The Your.MD Covid-19 symptom mapper's design and questions were selected in consultation with clinical experts. The symptom mapper allows participants to complete a survey on COVID-19 symptoms, any outcome of COVID-19 testing and recording underlying conditions as well as pre-specified risk factors for COVID-19. All survey questions are laid out in Supplementary Table 1.

Participants were invited to take part in Your.MD symptom mapping in a number of ways. Advertisements were run across the social media channels such as the Facebook network (Facebook, Instagram & Facebook Audience Network), Twitter, LinkedIn as well as the Your.MD webpage and app. These advertisements targeted both males and females aged 18+ globally but included a heavier geographic focus in: United Kingdom, United States, India, Nigeria, Bangladesh, Pakistan, Chile, Colombia, Mexico, Peru, Venezuela, El Salvador, Ecuador, Dominican Republic, and Bolivia. Advertisements used several formats including static images, carousels and videos that requested people to support in mapping the spread of COVID-19. The complete dataset of responders was available to the study team for analysis. As of 22nd September 2020, 175,566 people around the world used the mapper to record their symptoms.

### Data curation

This study made use of the Your.MD symptom mapper data ^7^ collected globally between 09/04/2020 and 22/09/2020. Of the 175,566 total responders to the survey, we chose those 78,299 responders for further analysis who were in the age range 0-100 years and had provided one of the following three responses as the reason for participating in the survey: ‘tested positive’ (n=7980), ‘tested negative’ (n=5620), or ‘showing symptoms but not tested’ (n= 64,699).  Responders who provided other reasons for participation in the survey such as ‘curious’, ‘self-isolating with no symptoms’, ‘live with someone with coronavirus’ were excluded from the analysis. All symptoms were mapped to binary categorical variables for analysis.

The symptoms of loss of appetite, chest pain, itchy eyes and joint pain were added 20 days later in the survey on 29/04/2020. This resulted in missing data for these symptoms for the 35,490 respondents (20.2% of the total) including 218 (2.7% of the total 7980) tested positive respondents. The missing data was excluded in the analysis of these symptoms.

### Statistical analysis

Characteristics of the responding cohort were described and visualised using histograms and heatmaps. Normalised frequency counts of symptoms were described and plotted, stratified by age and underlying chronic disease. Radar plots were used for visual comparison of symptom profiles and visualise differences between profiles. A comparative analysis was conducted to examine the association between symptoms, and groups of symptoms with geographical location of respondents and underlying chronic diseases reported. All comparisons were done using percentages or proportions (instead of the frequency counts) and the percentages/proportions for the symptoms added later were calculated after excluding the missing data. We compared the proportions using two-sided Chi-squared (χ2) test at a significance level of 5% with Benjamini-Hochberg correction applied to all the χ2 tests done in the analysis. All p values reported in the results are the corrected p values.

To assess the association between the presence of a symptom in a tested positive individual, the underlying chronic disease and the country of residence, a multivariable logistic regression model including *a priori* specified confounding variables of age and gender was developed for each symptom. Confounding variables were specified a priori based on clinical acumen and literature review.^10^ Age was converted to a binary variable (Age greater than 60 years was coded as 1).  The cut-off of 60 years to distinguish the older population was in line with the WHO report that the people over 60 years are at a higher risk of getting severe COVID-19 disease.^11^ The gender variable was coded as 0 for male and 1 for female. Country of residence was coded as a categorical variable. For the multivariable logistic regression analysis, data from the countries with more than 100 tested positive responders (12 countries discussed in Figure 4) was used, considering under-60-year male with no underlying chronic disease from Mexico (the country with the highest number of tested positive individuals) as reference. For the logistic regression, 95% confidence interval for adjusted odds ratio was calculated and variables with p-value ≤ 0.05 were considered as statistically associated with the presence of the symptom.

To understand the similarity between the symptom profile of the different chronic diseases and countries (Figure 3b and Figure 4b), we conducted a cluster analysis using agglomerative hierarchical clustering of the mean symptom profiles. Unlike other clustering methods, hierarchical clustering does not require a predefined number of clusters. To cluster the different chronic disease groups and countries based on their mean symptom profiles (Figures 3b and 4b), we used the Euclidean distance and the unweighted average distance as the linkage criteria. To cluster the symptoms for the different respondents of a chronic disease group or a country (Supplementary Figure 1 and 2), we used agglomerative hierarchical clustering with the Jaccard dissimilarity measure as the distance and unweighted average distance as the linkage criteria. The Jaccard dissimilarity measure is defined as one minus the Jaccard coefficient, which is the percentage of nonzero symptoms that differ.^12^ For clustering, we excluded symptoms experienced by less than 5% of the individuals in each group to avoid chaining.^13^ All statistical analyses were performed using custom programs in the MATLAB R2019b (MathWorks) environment.

### Ethics

The COVID-19 Symptom Mapper data was provided to us by Your.MD free of charge and no obligations with freedom to publish any results. The data is provided on request for free from Your.MD. On the Your.MD website all participants provided informed consent at the start of the online questionnaire for their data to be used for research purposes and had to agree to the corresponding Your.MD privacy and data usage policies. As the study used secondary data that were anonymised and obtained from Your.MD as a publicly available dataset, no ethical approval was sought.

We adhered to the Equator network - good practice in conduct and reporting of survey research where appropriate as we are making secondary use of data, and did not have a role in survey design.^14^ A statistical analysis plan and protocol were developed internally, but unpublished. As this was an exploratory study, we don't imply causality in our results.

### Role of the funding source

The funders had no role in study design or writing. All authors had full access to all the data in the study. The authors had sole responsibility for the decision to submit for publication.

## Results

### Characteristics of responders

**Figure 1:**
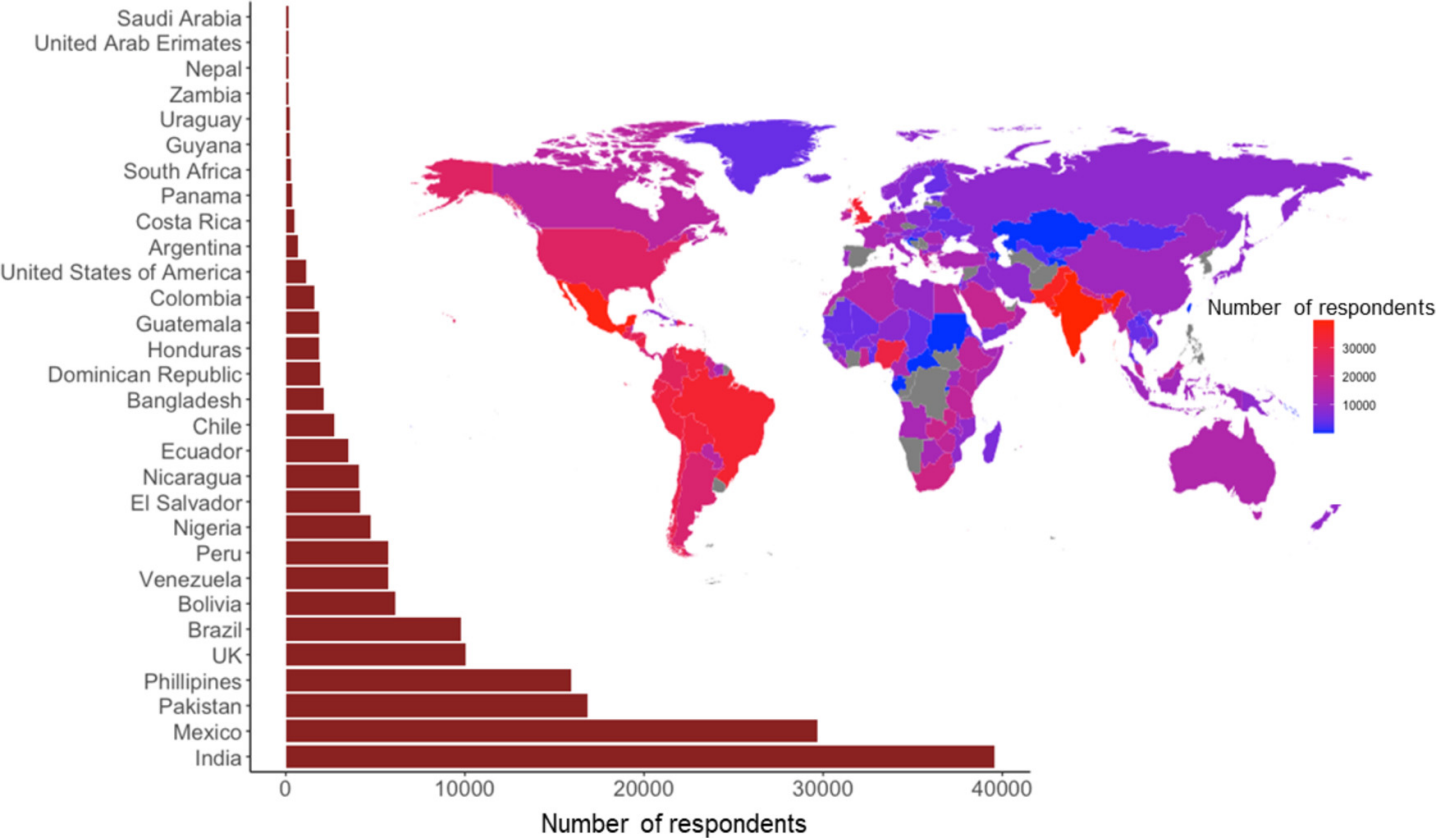
Global response to COVID-19 symptom survey. The bar chart shows the respondent counts for the top-30 countries with highest number of respondents. The map shows the heatmap of the respondent counts for all countries.

175,566 individuals from 190 different countries responded to the COVID-19 YOUR.MD questionnaire between 09/04/2020 and 22/09/2020. The countries with the highest proportion of respondents (Figure 1) were India (39590, 22.5%), Mexico (29644, 16.8%), Pakistan (16820, 9.5 %), Philippines (15950, 9.0 %), United Kingdom (10044, 5.7%), and Brazil (9796, 5.5%), accounting for 63.8 % of the total number of responders. The number of respondents for each of the 190 countries as Supplementary Table 2. The top-6 countries with the highest percentage of individuals who tested positive were Mexico (2199, 27.6%), Brazil (1366, 17.1%), Pakistan (753, 9.4%), India (714, 8.9%), United Kingdom (427, 5.3%), and Peru (427, 5.3%). Six other countries (Bolivia, Chile, Ecuador, Guatemala, Honduras, and Dominican Republic) had more than 100 tested positive respondents.

Data from responders with unknown COVID-19 status (e.g., ‘self-isolating with no symptoms’, ‘curious’, ‘live with someone with corona virus’), were excluded from the analysis, resulting in 78,299 respondents included in the analysis Table 1. describes the characteristics of responders who reported: a COVID-19 positive test; a COVID-19 negative test result, or that they were showing COVID-19 symptoms but not tested for all countries and the 6 countries with the most tested positive respondents. The statistics for the next 6 countries with the tested positive responders are available in Supplementary Table 3. Of these responders 7980 (10.2 %) tested positive, 5620 (7.2%) tested negative, and 64699 (82.6 %) reported symptoms but had not been tested. In the overall cohort mean age was 35.9 years (SD=11.6), responders who tested positive and negative for COVID-19 were older than responders who reported symptoms but had not been tested. A higher proportion of women completed the survey (59.3%). There was a higher proportion of health care workers who had been tested and reported a COVID-19 positive or COVID-19 negative test compared with those responders who were showing symptoms (18.1% vs 7.7%). Responders had been experiencing COVID-19 symptoms for a median of 4 days, with responders who were COVID-19 positive reporting a longer duration of symptoms (median, 7 days) compared with responders who had symptoms but had not been tested (median, 4 days).

**Table 1:** Characteristics of responders to Your.MD symptom questionnaire, categorised as tested positive, showing symptoms (but not tested), or tested negative. The results are presented as count(percentage) for binary characteristics and as mean (standard deviation) / median (inter quartile range) for continuous characteristics (age and number of days of symptoms). Statistical testing for untested symptomatic and tested negative groups were done in comparison to the tested positive group.

	Tested Positive n(%)	Untested Symptomatic n(%), p-value	Tested Negative n(%), p-value	All Responders n(%)
**All countries**	7980	64699	5620	78299
Age [in years] mean(SD)	38.9(11.5)	35.3(11.6), 1e-13	37.7(11.8), 4e-8	35.9(11.6)
Females	4993(62.6)	38183(59.0), 3e-8	3275(58.3), 1e-5	46451(59.3)
Pregnant	137(1.7)	915(1.4), 0.43	85(1.5), 2.77	1137(1.5)
Care home worker	725(9.1)	5621(8.7), 2.03	579(10.3), 0.24	6925(8.8)
Health care worker	1438(18.0)	4896(7.6), 1e-13	1020(18.1), 5.26	7354(9.4)
Number of days of symptoms mean(SD)	7.9(7.3)	6.1(7.6), 1e-13	6.3(8.0), 1e-13	6.3(7.6)
Number of days of symptoms median(IQR)	7.0(7.0)	4.0(5.0), 1e-13	4.0(6.0), 1e-13	4.0(5.0)
**Brazil**	1366	5089	802	7257
Age [in years] mean(SD)	40.6(10.9)	37.5(11.3), 1e-13	40.0(11.3), 1.96	38.4(11.3)
Females	1133(82.9)	4127(81.1), 1.20	649(80.9), 2.03	5909(81.4)
Pregnant	17(1.2)	67(1.3), 5.23	8(1.0), 3.99	92(1.3)
Care home worker	51(3.7)	175(3.4), 3.98	39(4.9), 1.81	265(3.7)
Health care worker	241(17.6)	476(9.4), 1e-13	151(18.8), 3.57	868(12.0)
Number of days of symptoms mean(SD)	8.4(7.0)	4.3(4.6), 1e-13	6.3(6.9), 9e-10	5.3(5.6)
Number of days of symptoms median(IQR)	8.0(7.0)	3.0(4.0), 1e-13	5.0(6.0), 9e-10	4.0(5.0)
**India**	714	7552	701	8967
Age [in years] mean(SD)	38.1(12.4)	34.1(11.4), 1e-13	34.4(11.7), 5e-7	34.4(11.5)
Females	282(39.5)	2901(38.4), 3.88	232(33.1), 0.18	3415(38.1)
Pregnant	21(2.9)	115(1.5), 0.06	14(2.0), 2.15	150(1.7)
Care home worker	121(16.9)	838(11.1), 6e-5	119(17.0), 5.72	1078(12.0)
Health care worker	84(11.8)	448(5.9), 3e-8	102(14.6), 1.20	634(7.1)
Number of days of symptoms mean(SD)	6.7(7.4)	7.4(9.8), 0.75	7.5(10.9), 0.90	7.3(9.7)
Number of days of symptoms median(IQR)	5.0(8.0)	4.0(5.0), 0.75	3.0(7.0), 0.90	4.0(6.0)
**Mexico**	2199	17432	985	20616
Age [in years] mean(SD)	38.5(10.6)	35.5(10.7), 1e-13	37.9(10.8), 1.59	35.9(10.8)
Females	1385(63.0)	11156(64.0), 2.77	597(60.6), 1.81	13138(63.7)
Pregnant	26(1.2)	211(1.2), 5.5	7(0.7), 1.97	244(1.2)
Care home worker	132(6.0)	990(5.7), 3.75	74(7.5), 1.12	1196(5.8)
Health care worker	357(16.2)	1194(6.8), 1e-13	155(15.7), 4.70	1706(8.3)
Number of days of symptoms mean(SD)	8.0(6.7)	5.1(5.4), 1e-13	5.2(5.5), 1e-13	5.4(5.6)
Number of days of symptoms median(IQR)	7.0(7.0)	4.0(5.0), 1e-13	4.0(5.0), 1e-13	4.0(5.0)
**Pakistan**	753	6286	412	7451
Age [in years] mean(SD)	36.1(12.2)	31.3(10.6), 1e-13	33.0(10.5), 2e-4	31.9(10.8)
Females	283(37.6)	2934(46.7), 4e-5	174(42.2), 1.20	3391(45.5)
Pregnant	29(3.9)	188(3.0), 1.78	13(3.2), 3.75	230(3.1)
Care home worker	119(15.8)	1244(19.8), 0.13	84(20.4), 0.60	1447(19.4)
Health care worker	160(21.2)	588(9.4), 1e-13	103(25.0), 1.37	851(11.4)
Number of days of symptoms mean(SD)	6.4(5.7)	4.8(6.2), 2e-9	4.7(5.9), 3e-5	5.0(6.1)
Number of days of symptoms median(IQR)	5.0(6.0)	3.0(4.0), 2e-9	3.0(5.0), 3e-5	3.0(4.0)
**United Kingdom**	427	2447	181	3055
Age [in years] mean(SD)	47.0(9.8)	47.5(13.6), 3.69	46.9(12.4), 5.26	47.4(13.1)
Females	348(81.5)	1864(76.2), 0.22	139(76.8), 1.69	2351(77.0)
Pregnant	1(0.2)	24(1.0), 1.23	1(0.6), 3.73	26(0.9)
Care home worker	110(25.8)	173(7.1), 1e-13	24(13.3), 0.01	307(10.0)
Health care worker	197(46.1)	316(12.9), 1e-13	65(35.9), 0.27	578(18.9)
Number of days of symptoms mean(SD)	10.1(8.4)	10.1(11.3), 5.72	12.1(13.1), 0.35	10.2(11.0)
Number of days of symptoms median(IQR)	8.0(8.0)	5.0(12.0), 5.72	6.0(17.0), 0.35	6.0(11.0)
**Peru**	427	2618	545	3590
Age [in years] mean(SD)	39.9(12.0)	37.6(12.0), 0.01	40.6(11.9), 2.56	38.3(12.1)
Females	233(54.6)	1567(59.9), 0.50	296(54.3), 5.57	2096(58.4)
Pregnant	7(1.6)	32(1.2), 3.51	8(1.5), 5.22	47(1.3)
Care home worker	32(7.5)	197(7.5), 5.72	49(9.0), 3.06	278(7.7)
Health care worker	43(10.1)	135(5.2), 0.001	68(12.5), 2.06	246(6.9)
Number of days of symptoms mean(SD)	7.5(8.0)	6.3(7.3), 0.02	5.5(5.7), 1e-4	6.3(7.2)
Number of days of symptoms median(IQR)	5.0(8.0)	4.0(5.0), 0.02	4.0(5.0), 1e-4	4.0(5.0)

### Symptoms reported

Table 2 describes symptoms reported by COVID-19 positive responders. Responders who tested positive were more likely to report joint pain (7.2% vs 4.9%, p<7e-9), loss of appetite (13.4% vs 7.5%, p<1e-13), and loss of smell and taste (44.6% vs 31.0%, p<1e-13) than responders who had tested negative or had symptoms but had not been tested. Fewer responders who had tested positive for COVID-19 reported sore throat (30.2% vs 44.2%, p<1e-13) and nasal congestion (33.0% vs 39.8%, p<1e-13) as symptoms compared with who had tested negative or had symptoms but untested. Interestingly, none of the responders selected sneezing as a symptom. Hence sneezing is not presented further in the results. Of responders who were COVID-19 positive, 60.3% had no underlying chronic disease, 30.7 % had one underlying chronic disease, 7.1% reported 2 underlying chronic diseases, and 1.9% reported 3 or more. The presence of underlying chronic disease was similar in the COVID-19 positive responders compared with the overall responder population. The detailed descriptive statistics for the individual countries and comorbidities are listed in Supplementary Table 4 and 5, respectively.

**Table 2:** Symptoms and comorbidities reported by Your.MD questionnaire responders from all countries. The results are presented as count(percentage).

	SARS-COV-2 status	
	All Responders n(%)	Tested Positive n(%), p-value
**Answered questions on chronic condition**	78299	7980
Reported one or more chronic conditions	29149(37.2)	3167(39.7), 2e-4
Asthma	3993(5.1)	417(5.2), 2.53
Diabetes type I	803(1.0)	117(1.5), 0.002
Diabetes type II	3161(4.0)	438(5.5), 8e-9
Heart disease	941(1.2)	104(1.3), 1.88
Hypertension	9668(12.3)	1139(14.3), 6e-6
Kidney disease	506(0.6)	43(0.5), 1.14
Liver disease	1416(1.8)	162(2.0), 0.79
Lung condition	568(0.7)	59(0.7), 3.45
Obesity	15681(20.0)	1618(20.3), 2.51
**Answered questions on symptoms**	78299	7980
Chest pain	3354(4.6)	552(7.1), 1e-13
Chills	15424(19.7)	1425(17.9), 5e-4
Cough	32762(41.8)	3283(41.1), 1.08
Diarrhoea	15802(20.2)	1980(24.8), 1e-13
Fatigue	37920(48.4)	4108(51.5), 2e-6
Headache	37826(48.3)	3640(45.6), 3.3e-5
Itchy eyes	11000(15.0)	1218(15.7), 0.69
Joint pain	4038(5.5)	562(7.2), 7e-9
Loss of Appetite	6291(8.6)	1043(13.4), 1e-13
Loss of smell and taste	25331(32.4)	3563(44.6), 1e-13
Muscle Ache	29234(37.3)	2738(34.3), 1e-6
Nasal Congestion	30635(39.1)	2635(33.0), 1e-13
Nausea and vomiting	4040(5.2)	449(5.6), 0.41
Shortness of breath	12975(16.6)	1248(15.6), 0.19
Sore throat	33479(42.8)	2413(30.2), 1e-13
Sputum	12057(15.4)	937(11.7), 1e-13
Temperature	16704(21.3)	1525(19.1), 2e-5

**Figure 2:**
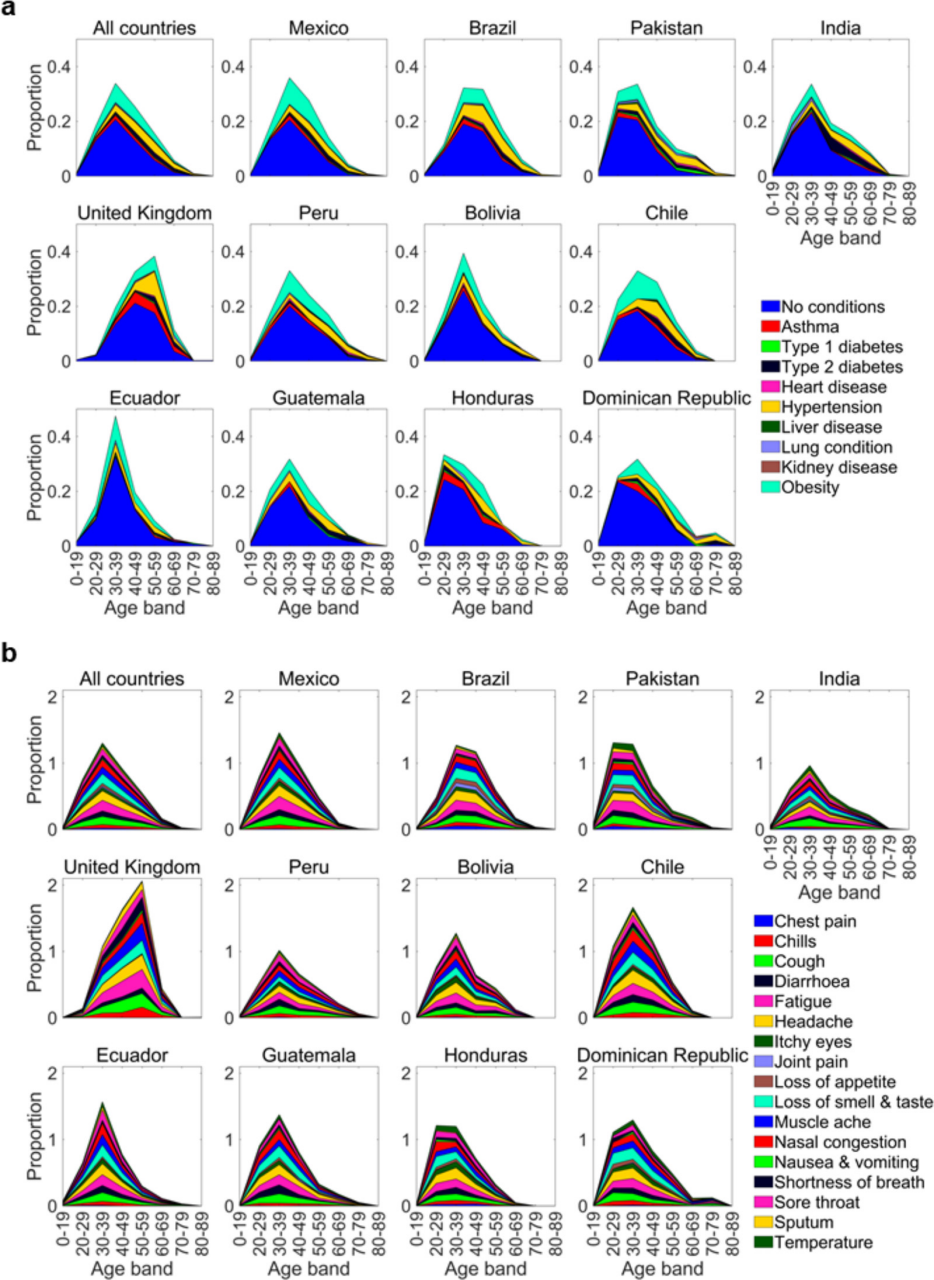
Age distribution of the population reporting underlying chronic diseases (a) and symptoms (b) of COVID-19 tested positive responders from the entire cohort and the top 12 countries. The proportions are counts as a fraction of the total number for that country. A respondent is counted once for the occurrence of each symptom and chronic disease.

Figure 2a shows the age distribution of the proportion of responders with and without a chronic disease who were COVID-19 positive, for all countries and the selected 12 countries with more than 100 tested positive responders Figure 2b shows the age distribution of the symptoms for individuals who were COVID-19 positive, for all countries and the selected 12 countries. The age profile of the responding population was centred around 20–29-year-old age band. Responders from the UK were on average older than other countries (47.0 years vs 38.9 years for all respondents, p=0.00001). Chile had the highest prevalence of comorbidities particularly obesity (27.1% vs 20.3% for all respondents, p=0.0022) compared with other represented countries.

### Symptom profiles by underlying chronic diseases

**Figure 3:**
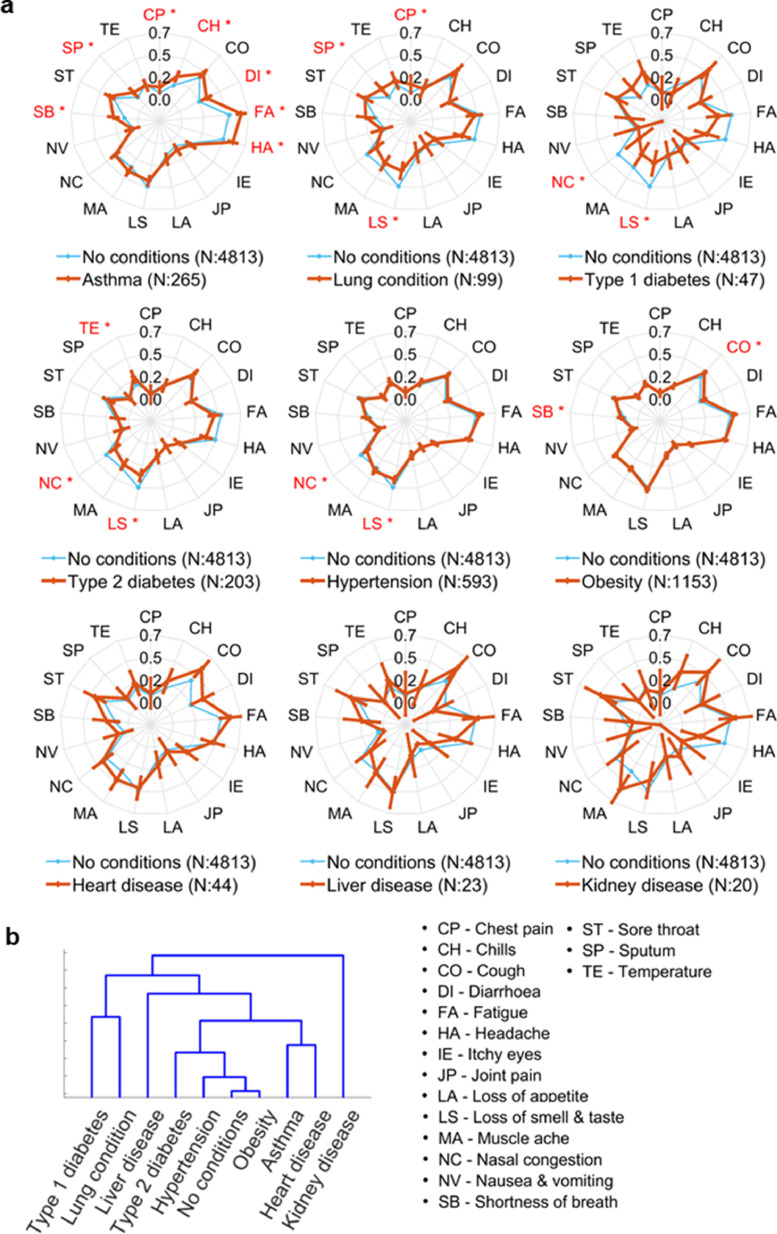
a. Visualisation of the proportion of tested positive responders having the symptoms impending on a pre-existing chronic disease (in red) against the base line of no underlying disease (blue). The histogram is represented as a radar plot where the 17 different symptoms are plotted along the 17 different radii of the circular radar plot. The further away a data point in the radar plot, the higher the frequency of the symptom. b. Cluster analysis of the mean symptom profile of the different chronic disease groups. The shorter the lines between the joining of the different chronic disease groups in the dendrogram, the smaller the difference between their mean symptom profiles.

To further explore the difference in symptom profiles associated with underlying chronic disease and location, a series of radar plots were produced to visualise multivariate data (Figure 3a). We are visualising the different complex patterns in symptoms depending on the condition of the underlying chronic disease condition using radar plots, to facilitate visual recognition of the difference in the patterns Figure 3a shows these symptom profiles amongst COVID-19 positive responders with and without underlying chronic disease. To better understand the impact of specific underlying chronic diseases, we have included only the responders with a single chronic disease for each disease to compare against the group with no chronic diseases. Differences in symptom profile between those with an underlying disease, and those without were evident across all comorbidity groups. For example, COVID-19 positive responders with asthma were more likely to report fatigue (62.3% vs 49.8%, p=0.003) headache (56.2% vs 45.8%, p=0.024), shortness of breath (25.3% vs 13.7%, p=7e-6), sputum production (17.4% vs 10.7%, p=0.023), chest pain (12.4% vs 6.8%, p=0.019), chills (26.8% vs 17.1%, p=0.002), or diarrhoea (31.3% vs 22.7%, p=0.031), compared with COVID-19 responders who had no underlying disease. COVID-19 positive responders with an underlying lung condition were more likely to report chest pain (15.2% vs 6.8%, p=0.038) and sputum (22.2% vs 10.7%, p=0.010) as a symptom, but less likely to report loss of smell and taste (29.3% vs 46.0%, p=0.025) compared with COVID-19 positive responders who had no underlying disease. Amongst COVID-19 positive responders with Type 2 diabetes, a raised temperature was more likely to be reported (26.1% vs 17.6%, p=0.045). Loss of smell and taste was more likely to be reported as a symptom amongst COVID-19 positive responders with no underlying disease (46.0%) compared with responders who had a lung condition (29.3%, p=0.025), Type 1 diabetes (21.3%, p=0.020), Type 2 diabetes (33.5%, p=0.014), or hypertension (37.8%, p=0.005).

We conducted a cluster analysis to understand the similarity between the symptom profile of the different chronic diseases (Figure 3b). In the clustering procedure, the mean symptom profiles of the different diseases were sequentially grouped according to the similarity of the symptom profile. In the dendrogram, the longer the lines between the joining of the different chronic disease groups, the greater the difference between their mean symptom profiles. The clustering found that the symptom profile of the responders with no chronic diseases was similar to the obese responders and this sub-group is clustered with type 2 diabetes and hypertension. The cophenetic correlation coefficient (a measure of how faithfully the clustering tree represents the similarities among observations) for the hierarchical cluster tree was 0.8 suggesting good clustering.

### Symptom profiles by countries

**Figure 4:**
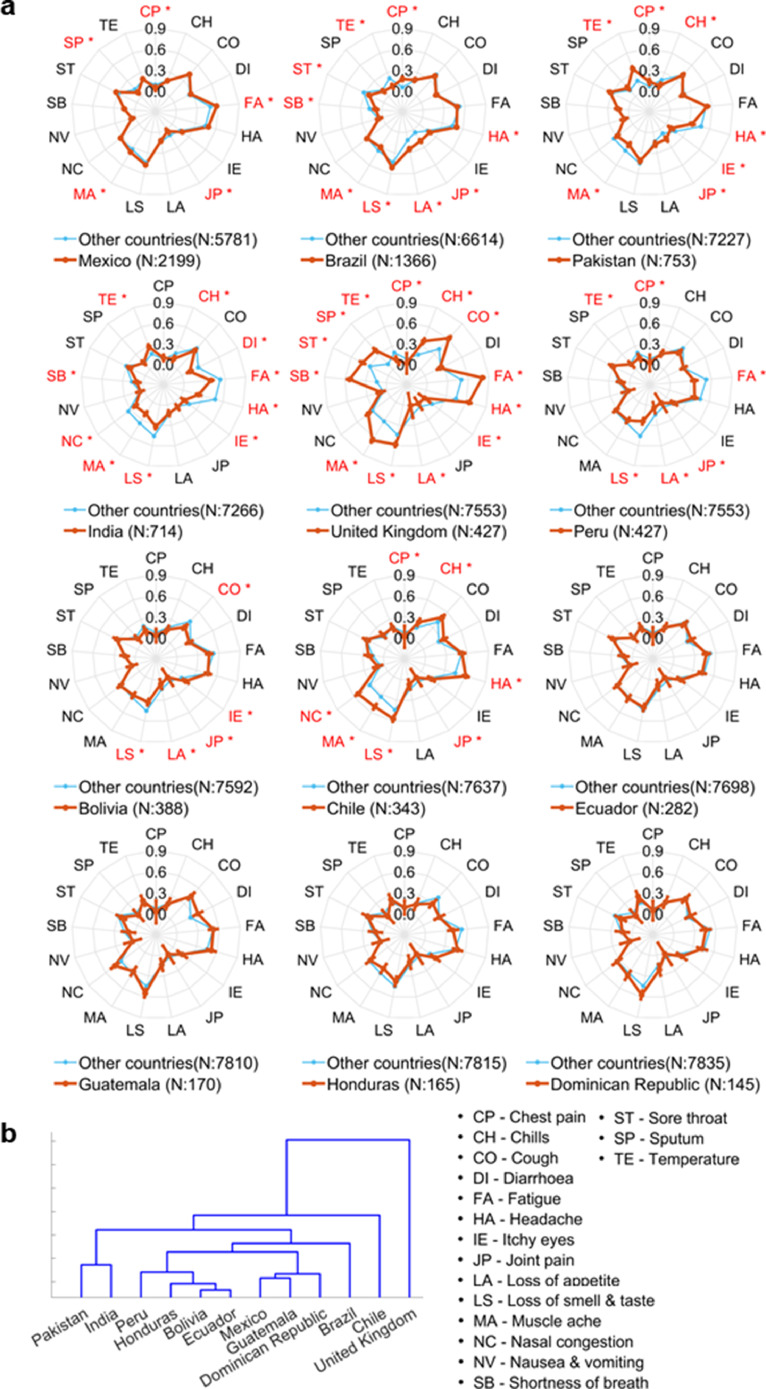
a. Visualisation of the symptom profiles of tested positive responders from different countries as radar plots. The 17 different symptoms are plotted along the 17 different radii of the circular radar plot. The further away a data point in the radar plot, the higher the frequency of the symptom. Error bars shown are standard errors and * denotes statistically significant difference (p<0.05, two-sided χ2 test). b. Cluster analysis of the mean symptom profile of the different countries. The shorter the lines between the joining of the different countries in the dendrogram, the smaller the difference between their mean symptom profiles.

Figure 4 shows differences in symptom profile from COVID-19 positive responders across countries. Brazil and Mexico reported a higher number of COVID-19 positive responders with itchy eyes (19.0% vs 15.0%, p=0.008 and 17.9% vs 14.8%, p=0.024) and headache (50.6% vs 44.6%, p=0.002 and 48.8% vs 44.4%, p=0.014). India and Pakistan reported fewer responders COVID-19 positive with nasal congestion (21.1% vs 34.2%, p=2e-10 and 27.5% vs 33.6%, p=0.020) and muscle ache (19.5% vs 35.8%, p<1e-13 and 25.1% vs 35.2%, p=1e-6) compared with other countries. Chile (7.3% vs 13.7%, p=0.019) and Bolivia (6.7% vs 13.8%, p=0.003) reported higher number of COVID-19 positive responders with loss of appetite. A higher number of COVID-19 positive responders in Brazil reported itchy eyes (19.0% vs 15.0%, p=0.008), loss of smell and taste (50.8% vs 43.4%, p=2e-5), appetite loss (24.2% vs 11.1%, p<1e-13), joint pain (19.1% vs 4.7%, p<1e-13), headache (50.6% vs 44.6%, p=0.002), and chest pain (16.8% vs 5.0%, p<1e-13) compared with other countries. A higher number of COVID-19 positive responders in India (28.8% vs 18.2%, p=3e-10) reported a high temperature as a symptom, compared with other countries. In Mexico, a higher number of COVID-19 positive responders reported muscle ache (37.8% vs 33.0%, p=0.002), itchy eyes (17.9% vs 14.8%, p=0.024), fatigue (57.7% vs 49.1%, p=4e-10), sore throat (32.8% vs 29.3%, p=0.049) and headache (48.8% vs 44.4%, p=0.014) compared with other countries. A higher number of COVID-19 positive responders in Pakistan reported a high temperature (35.9% vs 17.4%, p<1e-13), joint pain (15.6% vs 6.3%, p<1e-13) or chest pain (12.9% vs 6.5%, p=9e-9) compared with other countries. Differences were most marked for COVID-19 positive responders from the UK. A higher number of responders reported loss of smell and taste (58.1% vs 43.9%, p=6e-7), muscle ache (64.9% vs 32.6%, p<1e-13), shortness of breath (53.4% vs 13.5%, p<1e-13), sputum (37.5% vs 10.3%, p<1e-13), sore throat (41.7% vs 29.6%., p=6e-6), chills (37.7% vs 16.7%, p<1e-13), cough (61.4% vs 40.0%, p<1e-13), fatigue (80.6% vs 49.8%, p<1e-13), and headache (64.2% vs 44.6%, p=3e-13) compared with other countries. This may reflect the differing age range of the UK responder population. In Peru, a lower number of COVID-19 positive responders reported chest pain (1.6% vs 7.4%, p<1e-3), fatigue (34.9% vs 52.4%, p=2e-10), headache (37.7% vs 46.1%, p=0.021), joint pain (0.9% vs 7.6%, p=1e-5), loss of smell and taste (23.9% vs 45.8%, p<1e-13), appetite loss (4.0% vs 13.9%, p=2e-7) and temperature (11.9% vs 19.5%, p=0.004) compared with other countries.

Figure 4b shows the cluster analysis of the mean symptom profile of the different countries. In the clustering procedure, the mean symptom profiles of the different countries were sequentially grouped according to the similarity of the symptom profile. The shorter the lines between the joining of the different countries in the dendrogram, the smaller the difference between their mean symptom profiles. It is interesting to note that the clustering algorithm has grouped the countries mostly by geographical location though only the symptom profile and no geographical information was input to the algorithm. Pakistan and India are in one cluster, the three south American countries of Peru, Ecuador and Bolivia are in another cluster, Mexico is in the same cluster as Guatemala and Dominican Republic, and the United Kingdom is in a separate cluster. This suggests that the geography is an important factor in the symptom profiles of the tested positive respondents. The cophenetic correlation coefficient for the hierarchical cluster tree was 0.9 suggesting very good clustering.

We ran a cluster analysis to understand how symptoms co-occur in different chronic disease groups and countries and the dendrogram of the clustering is presented in Supplementary Figure 1 and 2. The clustering of symptoms across COVID-19 positive responders shows that fatigue, muscle ache and headache were the most co-occurring symptoms.

### Multivariable analysis

**Figure 5:**
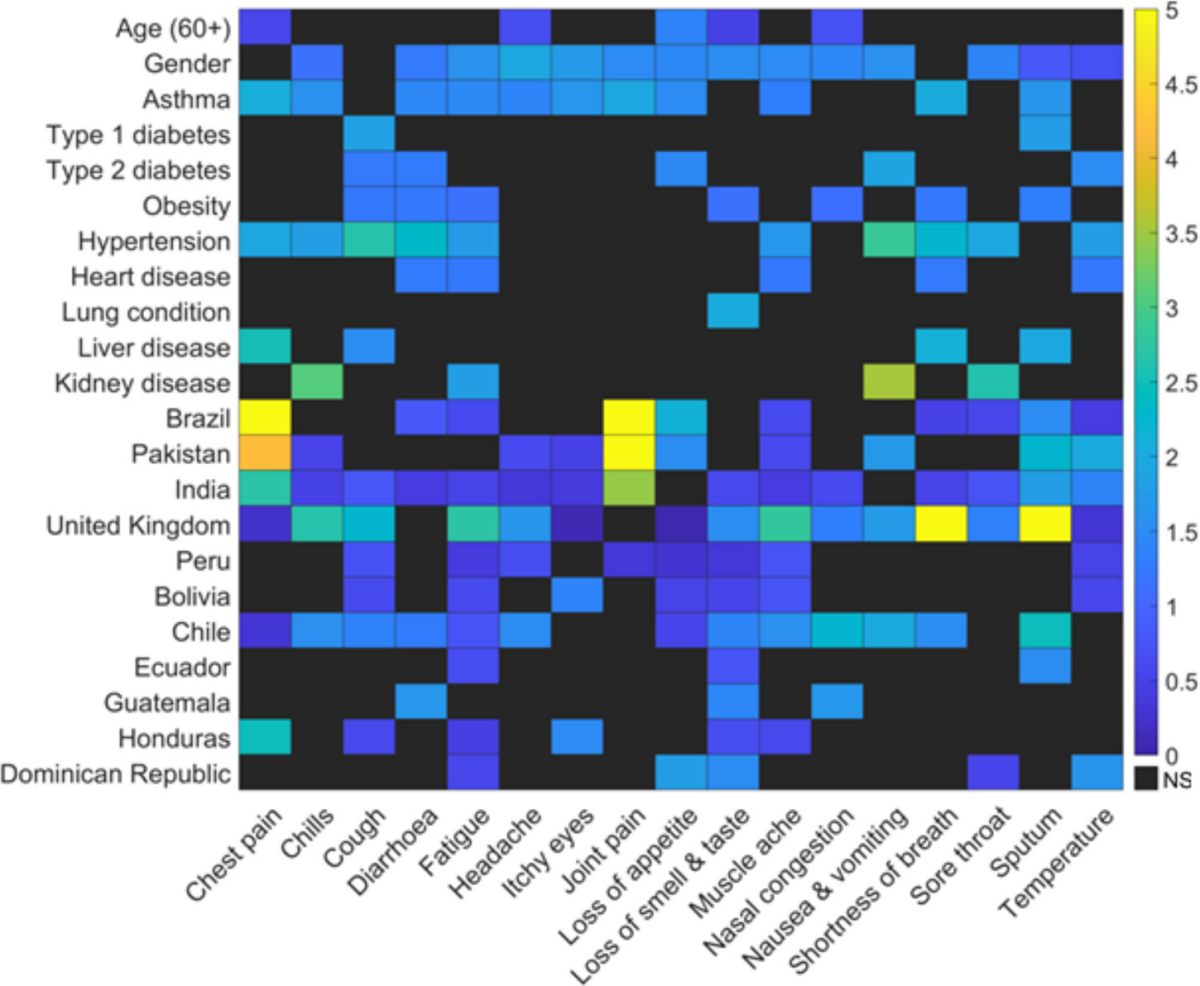
Association between symptoms, underlying chronic disease, countries, age, and gender in tested positive responders. Each column represents the adjusted odds ratio of the different factors (age, gender, chronic disease, and countries) in a multivariable model for the presence of the symptom corresponding to the column. Odds ratio which are statistically significant (p<0.05, 95% CI excludes 1) are coloured. Odds ratio which are not statistically significant are marked as NS.

Symptoms profiles for COVID-19 positive patients varied based on underlying chronic disease and country. ​ To investigate the association between each of the 17 symptoms and the chronic disease and country of residence, a multivariable logistic regression model was developed for each symptom adjusting for age and gender. The results are presented in Figure 5 with the adjusted odds ratio (AOR) colour coded to indicate strength of association. Each column of Figure 5 shows the association between the explanatory variable (comorbidity and country of residence mentioned in the row) and the odds ratio of the presence of the symptom (mentioned in the column) in the tested positive responders. The model tables for the 17 logistic regression models built are available in Supplementary Table 6.

After adjustment for other chronic diseases, gender, age and country of residence, the odds of shortness of breath is higher for COVID-19 positive responders with asthma (AOR=2.02, 95% CI=(1.56 – 2.62), p=9e-8), hypertension (2.23 (1.36 – 3.66), p=0.001), liver disease (2.11 (1.4 – 3.2), p=0.0004) and United Kingdom (6.45 (5.12 – 8.11), p<1e-13) and lower for Brazil (0.5 (0.40 – 0.63), p=3e-9) and India (0.52 (0.39 – 0.70), p=2e-5). COVID-19 positive responders with asthma had higher odds of reporting chest pain (2.07 (1.47 – 2.93), p=3e-5) and sputum production (1.64 (1.23 – 2.20), p=0.0007). COVID-19 positive responders with hypertension had the greatest odds of reporting shortness of breath (2.23 (1.36-3.66), p=0.001), diarrhoea (2.31 (1.51 – 3.54), p=0.0001), or cough (2.66 (1.72 – 4.10), p=1e-5), compared with other chronic condition. COVID-19 responders from Brazil had the greatest odds of experiencing chest pain (5.4 (4.10 – 7.11), p<1e-13), joint pain (7.9 (5.88 – 10.63), p<1e-13), and loss of appetite (2.14 (1.78 – 2.57), p<1e-13) compared with other countries whilst controlling for age, gender, and chronic diseases.

## Discussion

This is the first study to explore symptoms among those who test positive for COVID-19 by geolocation and underlying chronic disease. We find that there are geographic and underlying disease symptom differences, and this understanding is crucial for clinical practice: to speed up diagnosis; enable more precise prediction of outcomes, and target treatment. Symptoms for COVID-19 positive patients varied based on underlying chronic disease and based on geographical location in both crude and adjusted logistic regression models.

The global impact of the COVID-19 pandemic has led to a rapid development and utilization of mobile health applications.^15^ To facilitate an agile response to the pandemic, self-reported survey responses on health, behaviour and demographics have been introduced in countries around the world to better understand symptom presentation. However, evidence suggests that their conclusions are highly variable. A recent Cochrane review found data on 84 signs and symptoms in 44 studies, but only 10 symptoms were reported by more than 10 studies. The top ten most often reported signs and symptoms were fever, cough, shortness of breath, sore throat, muscle soreness, diarrhoea, headache, fatigue, sputum production, and loss of smell or taste.^16^ An early meta-analysis of epidemiological variation in COVID-19 inside and outside China studied patient characteristics including, gender, age, fatality rate, and symptoms of fever, cough, shortness of breath and diarrhoea in COVID-19 patients. They found that important symptom differences existed in patients in China compared to other countries and recommended that clinical symptoms of COVID-19 should not be generalized to fever, shortness of breath and cough only, but other symptoms such as diarrhoea are also shown to be prevalent in patients with COVID-19.^17^ The use of symptom profiles to predict COVID-19 positive PCR has also had mixed results. A recent evaluation of the diagnostic accuracies of web-based COVID-19 symptom checkers found a variation in sensitivity and specificity of symptoms mappers meta-analysed. This was in part due to the wide variation in symptoms that could be added to individual symptom mappers.^1^

Responders from India comprised a high proportion of our study cohort and our study shows that amongst the Indian COVID-19 positive responders, fatigue, joint pain, muscle ache and headache were most commonly reported. A survey conducted in August 2020 in the Indian population aimed to assess knowledge and awareness on COVID-19.^18^ Questions on awareness about coronavirus symptoms revealed that considerable numbers of respondents acknowledged fever, and persistent cough as frequent symptoms of COVID-19, but relatively few responders could list additional symptoms. This highlights a lack of knowledge on the most prevalent symptoms within that country.

A recent Brazilian study aimed to analyse the profile of COVID-19 symptoms and related aspects. Using data from 346,181 individuals who completed the *Brazilian National Household Sample Survey* in May 2020.^19^ Eleven key symptoms were reported which were widely reported in the population: fever, cough, sore throat, difficulty breathing, headache, chest pain, nausea, stuffy or runny nose, fatigue, eye pain and loss of smell or taste. Female sex, brown skin colour, the North and Northeast regions of Brazil, and all three older age brackets showed stronger association with all the symptoms. Our results agree with the profile of symptoms reported in the Brazilian population, and we also observe age related increase in odds of symptoms, across all countries.

An observational study on 482,413 individuals who were tested for COVID-19 in Mexico found high incidence in working-age Mexican outpatients, the main symptoms among people tested were headache, arthralgia or myalgia, and sore throat. ^20^ Using clustering techniques 3 symptomatic profiles were suggested which grouped the 11 symptoms. These symptoms correspond to the symptoms recorded in our study. In our study Mexican responders with a COVID-19 positive result frequently reported fatigue, headache, itchy eyes, and sort throat. In addition, our study found that loss of smell and taste was reported more frequently in the Mexican COVID-19 positive responders than in responders from other countries.

Public health guidance in the UK advises that the triad of symptoms: new persistent cough; high temperature, loss of taste and smell.^21^ In the UK a national symptom tracker app collects data from both asymptomatic and symptomatic individuals and tracks in real time how the disease progresses by recording self-reported health information on a daily basis, including symptoms, hospitalization, reverse-transcription PCR (RT-PCR) test outcomes, demographic information and pre-existing medical conditions.^10^ A recent report based on 2,618,862 individuals who used the app identified a combination of symptoms, in addition to the more stablished symptoms, including anosmia, fatigue, persistent cough and loss of appetite, that together might identify individuals with COVID-19.^10^

These results were further affirmed by the UK REACT (REal-time Assessment of Community Transmission).^22^ In addition to previously reported symptoms which were predictive of COVID-19 positive PCR, the REACT programme reports that chills, headache, appetite loss and muscle aches should be added to the catalogue of COVID-19 symptoms.^22^ The study also found that there was a variation in symptoms with age. While chills were linked with testing positive across all ages, headaches were reported in young people aged five to 17 and appetite loss was reported more in 18-54 year-olds and those aged 55 and over. Cough was observed in two thirds of cases in a systematic review and the largest cohort studies, suggesting it is unreliable alone as a key diagnostic symptom.^23^ Our findings support these results, with fatigue, muscle ache, headache, cough, and loss of smell and taste being reported as prevalent symptoms in the UK responding population in our study. Muscle ache was more prevalent in COVID-19 positive responders in the UK compared with other countries in our study (64.9% in UK vs 32.6% in other countries, p<1e-13). In the REACT study muscle ache was mostly reported in people aged between 18 and 54. Our study supports this finding, and this may reflect the older demographic of the UK population within our study.

One of the strengths of this study was the ability to look globally at symptoms with a specific breakdown by nationality, allowing geolocation and culture/behavioural aspects to be investigated. It is possible that the differences reported globally come from the different perception of symptoms in different countries. Symptom reports were conducted in local languages (e.g., Portuguese, Hindi, etc) thus increasing accessibility, however translations may not match exactly within cultural contexts, e.g., “pain” in Brazil is “joint ache” in UK (but not stomach pain).

The cohort of web-based symptom mappers like our study may not represent a random sampling of the population. However, this is a limitation inherent to all epidemiologic studies relying on voluntary participation. While random sampling comes at the severe cost of the risk of shrinking a global survey's size and power, the results of surveys without random sampling are still valid measures for public health policymaking. Throughout the course of the pandemic, public health response has made use of data from opportunistic surveys and sampling of the population, such as the current study. The average age of a UK responder in our study was 47 years. A similar survey^10^ using data collected via an app in the UK used similar methods to invite participation, and showed an average responder age of 41 years, with a similar breakdown of male/female responders. Whilst all survey studies will be limited in terms of external validity, these sampling methods allow for rapid accumulation of data from the population. Both ours and the external study^10^ indicate that older members of the population were motivated to complete the survey. Crucially, the ongoing UK-based study REACT^22^ which directly informs the UK government examined symptoms in a randomly sampled population of one million patients and found results similar to ours, for example suggesting that chills, headache, appetite loss and muscle aches are associated with COVID PCR positive test, in divergence to the classically reported COVID symptom triad.

Given the data for this analysis came from an Internet based survey, there will be differential access, however only a very low effort was needed to partake given the questionnaire was accessed via a simple website and not an app. Given the widespread use of smartphones globally, this should facilitate participation, however we acknowledge that those who are younger or in wealthier countries may be more likely to partake thus skewing the results, equally educational factors may have played a role and we do not have any socioeconomic or ethnicity information. Also, no definition of the symptoms and the term “long-standing” in the long-term health conditions was provided in the survey which might have caused some interpretation issues. As the mapper was developed when Covid-19 only recently emerged and without extensive knowledge of the coronavirus, it was decided not to define the symptoms to enable open interpretations from respondents. Nevertheless, the wording of each question was carefully chosen to avoid dubiousness. Whilst we acknowledge that the data used are self-reported, we do not think this undermines the accuracy of underlying disease or symptom reporting. For those who report a COVID-19 positive test, we do not distinguish between type of tests and thus cannot account for differences in accuracy.

Our information may be utilised in a clinical setting as an additional triage tool and for target testing, especially to better inform decisions in patient groups with preconditions, co- and multi-morbidities, but also in countries where no published symptom profiles have been reported. Symptom checkers are being widely used in response to the global COVID-19 pandemic. A recent study reported that web-based COVID-19 symptom checkers vary widely in their predictive capabilities, with some performing equivalently to random guessing while others show strength in sensitivity, specificity, or both. (1)

The results have wider public health implications beyond direct clinical care. The study highlights the importance of looking beyond the classic public health messaging on the classic symptoms of COVID-19. Our study corroborates findings from individual countries (Please see Supplementary Table 7) that a wide variety of symptoms are associated with COVID-19. Public health messaging in many countries is focussed on advice to seek a COVID-19 test if the triad of symptoms cough, fever and loss of taste/smell are present or variations thereof. The results indicate that amongst those patients with a COVID-19 positive test, and regardless of underlying chronic disease the most frequently reported symptoms did not include cough, fever, and loss of taste/smell. Mismatches between the symptoms that populations were communicated to look out for and those they are actually presenting may mislead patients to assume an asymptomatic behavioural stance while they may be actually COVID-19 positive. If population testing around the world is triggered by symptom criteria that are inaccurate this could potentially bias any prevalence estimates, leading to an underrepresentation of COVID-19 positive cases, which will hamper measures to control and manage the pandemic.

### Contributions

Study concepts & design: BK, GB, JKQ, CEC, AAF

Literature search: CEC

Data cleaning: BK, GB

Statistical Analysis: BK, GB

Data Analysis: BK, GB, JKQ, CEC, AAF

Manuscript draft: All authors

Figure preparation: BK, GB

Final manuscript review: All authors

## Declaration of interests

JKQ reports grants from MRC, grants from GSK, grants and personal fees from AZ, grants and personal fees from BI, grants and personal fees from Chiesi, grants from The Health Foundation, grants from Bayer, grants from Asthma UK, outside the submitted work. Other authors declare no competing interests.

## Appendix Supplementary materials

Image, application 1

Image, application 2

Image, application 3
