# Supplementary material for: Variation in global COVID-19 symptoms by geography and by chronic disease: A global survey using the COVID-19 Symptom Mapper
Source: eClinicalMedicine. 2022 Mar 6;45:101317. doi: 10.1016/j.eclinm.2022.101317 (PMC8898170; doi:10.1016/j.eclinm.2022.101317)
Supplement: Supplementary file 3 [file mmc3.docx]

**Supplementary Material**


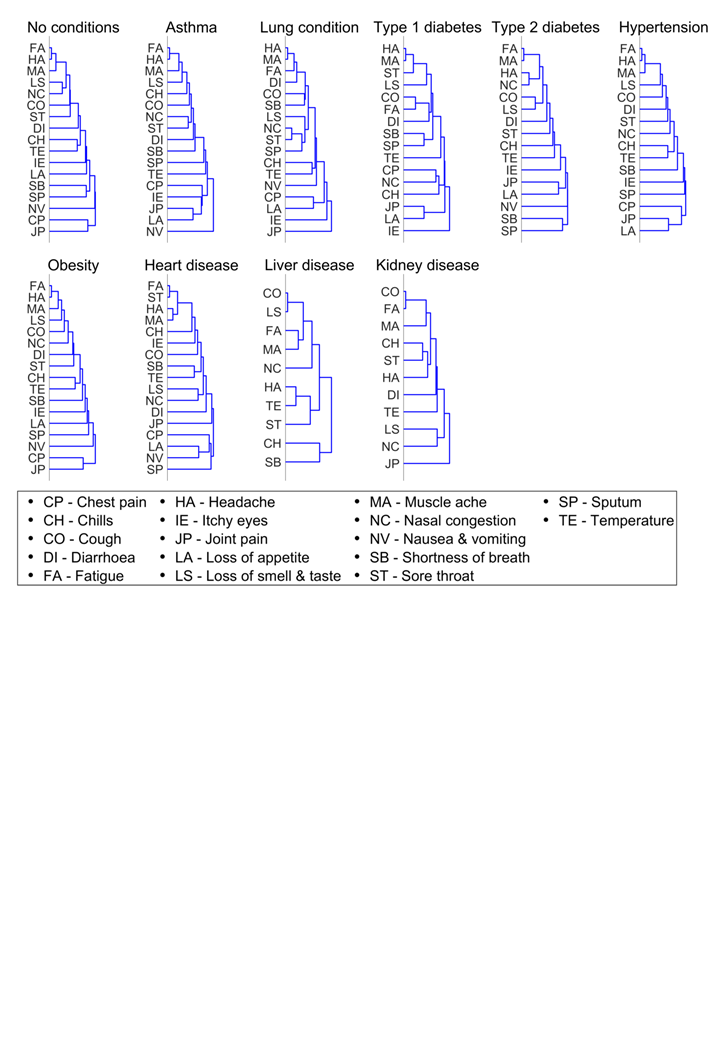


**Supplementary Figure 1 | Dendrograms of clustering of the symptoms for different countries amongst COVID-19 positive responders. The shorter the lines between the joining of the different symptoms in the dendrogram, the higher the co-occurrence of the symptoms.**


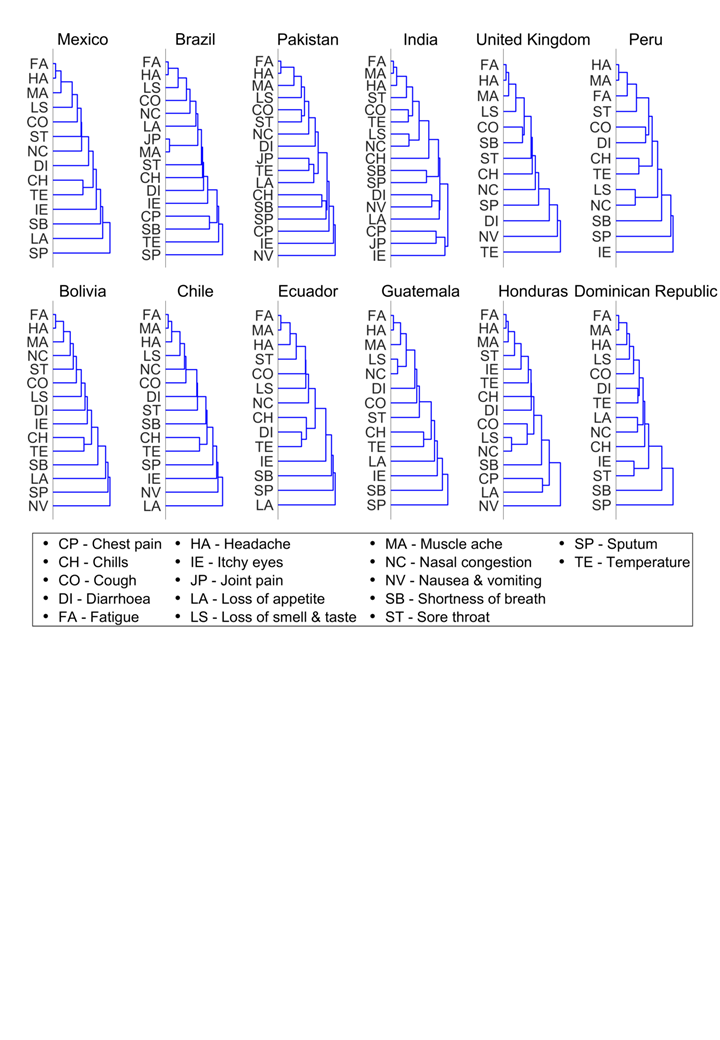


**Supplementary Figure 2| Dendrograms of clustering of the symptoms for different comorbidity groups amongst COVID-19 positive responders. The shorter the lines between the joining of the different symptoms in the dendrogram, the higher the co-occurrence of the symptoms.**

**Supplementary Table 1 | Survey questions asked at the YourMD symptom mapper webpage**

| **Questions** | **Options** |
| --- | --- |
| First of all, it's important to find out if you need urgent attention. Are you experiencing any of the following? | Severe shortness of breath - more than 25 breaths per minute  Pain or pressure in your chest  Cold clammy or pale and mottled skin  Feeling confused (if that’s new)  Coughing up blood  Blue lips or face  Sudden collapse or fainting  Difficulty waking up  Little or no urine output |
| What’s the main reason you’d like to map your symptoms? Please choose the most relevant reason only. | I’ve tested positive for coronavirus and have symptoms    I tested negative but now have symptoms    I have symptoms but haven’t been tested    I’ve recovered from coronavirus but have new symptoms    I’m just interested in seeing the symptoms comparison, I’ don’t have symptoms    I am isolating but have no symptoms |
| How are you feeling? | I feel fine    I feel sick    I feel very sick |
| How many days have you had symptoms? | From 0 to ‘more than 42’ |
| Now for the important bit. Do you have any of these symptoms? [Multiple Answer Tick Boxes] | Cough  Fever  Fatigue/tiredness  Headache  Blocked nose  Diarrhoea  Loss of appetite  Chest pain /tightness  Joint pain /aches  Coughing up mucus  Shortness of breath  Muscular aches  Sore throat  Chills  Vomiting  Loss of taste and smell  Sneezing  Itchy eyes |
| We are keen to understand how COVID-19 is affecting people with long-standing health problems. Do you have any of these conditions? [Multiple Answer Tick Boxes] | Long-standing lung condition    Asthma (managed with inhaler)    Long standing heart Disease    High Blood Pressure (hypertension)    Diabetes Type 1 (controlled by insulin)    Diabetes Type 2    Long-Standing Kidney Disease    Long Standing Liver Disease    Obesity |
| Take a look at these descriptions of mild, moderate and severe COVID-19. At the end, tell us which one best describes how you’re feeling now. | Mild COVID-19 How it feels: You’ll have flu-like symptoms, which can include cough, mild fever, headache, muscle pain and tiredness. You won’t be short of breath in general. You shouldn’t have a high fever. Keep yourself hydrated and rest. More info... Moderate COVID-19 How it feels: You’re more breathless and feel your heartbeat faster when you move around. The cough is more noticeable. You’ll be coughing many times an hour and it’s probably making you tired. You may have symptoms of a fever like shivering and chills. More info... Severe COVID-19 How it feels: You’re going to feel very breathless (even when you’re resting), and might find it hard to finish sentences. Along with chest, stomach or back pain when you breathe, these can be signs you’ve developed pneumonia. You will not be eating and drinking normally. Other signs include a high fever. More info...  Warning: If you’re feeling increasingly breathless, can’t manage basic things like showering and eating, or if you can’t speak in whole sentences without taking extra breaths, call your local health service right away.  *Read Dr Mary Lowth’s authoritative article on the stages of coronavirus on [Healthily](https://www.livehealthily.com/blog/coronavirus-whats-the-difference-between-mild-moderate-and-severe-illness) |
|  |  |
| After reading this, would you say your symptoms are | Mild; Moderate, Severe, None of the above |
| Town/City and Country |  |
| Age |  |
| Sex at birth | Male/Female |
| Are you pregnant? | Yes/No |
| Are you a healthcare worker? | Yes/No |
| Are you a care home worker? | Yes/No |

**Supplementary Table 2 | Count of survey respondents by country. The table is also added as an additional file along with the manuscript**

**Supplementary Table 3 | Characteristics of responders to Your.MD symptom questionnaire from Bolivia, Chile, Ecuador, Guatemala, Honduras, Dominican Republic, categorised as tested positive, showing symptoms (but not tested), or tested negative. The results are presented as count(percentage) for binary characteristics and as mean (standard deviation) / median (inter quartile range) for continuous characteristics (age and number of days of symptoms).**

| **SARS-CoV-2 status** | | | | |
| --- | --- | --- | --- | --- |
|  | **All responders**  **n(%)** | **Tested**  **Positive**  **n(%)** | **Untested Symptomatic**  **n(%)** | **Tested**  **Negative**  **n(%)** |
| **Bolivia** |  |  |  |  |
| Count | 4385 | 388 | 3639 | 358 |
| Age [in years]  mean(SD) | 35.2(10.5) | 37.7(10.4) | 34.8(10.5) | 37.0(10.1) |
| Females | 2447(55.8) | 234(60.3) | 2018(55.5) | 195(54.5) |
| Pregnant | 65(1.5) | 9(2.3) | 50(1.4) | 6(1.7) |
| Care home worker | 347(7.9) | 23(5.9) | 289(7.9) | 35(9.8) |
| Health care worker | 398(9.1) | 62(16.0) | 281(7.7) | 55(15.4) |
| Number of days of symptoms mean(SD) | 5.2(5.9) | 8.1(8.2) | 5.0(5.6) | 4.8(5.1) |
| Number of days of symptoms median(IQR) | 4.0(5.0) | 7.0(9.0) | 4.0(5.0) | 4.0(6.0) |
| **Chile** |  |  |  |  |
| Count | 1675 | 343 | 1202 | 130 |
| Age [in years]  mean(SD) | 36.3(10.9) | 37.2(10.3) | 36.1(11.1) | 35.7(10.2) |
| Females | 1236(73.8) | 248(72.3) | 904(75.2) | 84(64.6) |
| Pregnant | 27(1.6) | 5(1.5) | 19(1.6) | 3(2.3) |
| Care home worker | 68(4.1) | 16(4.7) | 45(3.7) | 7(5.4) |
| Health care worker | 156(9.3) | 63(18.4) | 78(6.5) | 15(11.5) |
| Number of days of symptoms mean(SD) | 5.4(5.6) | 7.2(5.9) | 4.7(5.0) | 6.6(8.0) |
| Number of days of symptoms median(IQR) | 4.0(5.0) | 6.0(5.0) | 3.0(4.0) | 4.0(5.0) |
| **Dominican Republic** |  |  |  |  |
| Count | 1244 | 145 | 956 | 143 |
| Age [in years]  mean(SD) | 34.9(11.3) | 37.7(13.0) | 34.2(10.7) | 36.9(12.6) |
| Females | 929(74.7) | 103(71.0) | 720(75.3) | 106(74.1) |
| Pregnant | 34(2.7) | 4(2.8) | 24(2.5) | 6(4.2) |
| Care home worker | 105(8.4) | 7(4.8) | 87(9.1) | 11(7.7) |
| Health care worker | 141(11.3) | 26(17.9) | 94(9.8) | 21(14.7) |
| Number of days of symptoms mean(SD) | 5.4(6.8) | 8.0(6.9) | 4.9(6.4) | 6.2(8.2) |
| Number of days of symptoms median(IQR) | 4.0(5.5) | 7.0(10.0) | 3.0(5.0) | 4.0(5.0) |
| **Ecuador** |  |  |  |  |
| Count | 2163 | 282 | 1596 | 285 |
| Age [in years]  mean(SD) | 34.0(10.8) | 36.7(10.3) | 33.1(10.5) | 36.3(11.6) |
| Females | 1236(57.1) | 171(60.6) | 913(57.2) | 152(53.3) |
| Pregnant | 26(1.2) | 6(2.1) | 16(1.0) | 4(1.4) |
| Care home worker | 205(9.5) | 25(8.9) | 147(9.2) | 33(11.6) |
| Health care worker | 158(7.3) | 30(10.6) | 85(5.3) | 43(15.1) |
| Number of days of symptoms mean(SD) | 6.7(8.4) | 9.1(8.6) | 6.5(8.5) | 5.8(6.9) |
| Number of days of symptoms median(IQR) | 4.0(6.0) | 7.5(9.0) | 4.0(5.0) | 4.0(6.0) |
| **Guatemala** |  |  |  |  |
| Count | 1217 | 170 | 985 | 62 |
| Age [in years]  mean(SD) | 34.9(10.9) | 38.2(11.7) | 34.4(10.6) | 34.6(11.4) |
| Females | 640(52.6) | 94(55.3) | 513(52.1) | 33(53.2) |
| Pregnant | 14(1.2) | 0(0.0) | 13(1.3) | 1(1.6) |
| Care home worker | 150(12.3) | 19(11.2) | 125(12.7) | 6(9.7) |
| Health care worker | 96(7.9) | 18(10.6) | 67(6.8) | 11(17.7) |
| Number of days of symptoms mean(SD) | 5.1(5.5) | 7.2(5.7) | 4.8(5.5) | 3.9(3.8) |
| Number of days of symptoms median(IQR) | 4.0(5.0) | 6.0(6.0) | 3.0(4.0) | 3.0(6.0) |
| **Honduras** |  |  |  |  |
| Count | 1168 | 165 | 808 | 195 |
| Age [in years]  mean(SD) | 33.5(10.8) | 34.6(10.5) | 33.0(10.9) | 34.8(10.6) |
| Females | 761(65.2) | 108(65.5) | 526(65.1) | 127(65.1) |
| Pregnant | 21(1.8) | 4(2.4) | 15(1.9) | 2(1.0) |
| Care home worker | 148(12.7) | 18(10.9) | 105(13.0) | 25(12.8) |
| Health care worker | 114(9.8) | 23(13.9) | 57(7.1) | 34(17.4) |
| Number of days of symptoms mean(SD) | 5.6(6.5) | 7.5(7.3) | 5.2(6.5) | 5.4(5.8) |
| Number of days of symptoms median(IQR) | 4.0(5.0) | 6.0(8.0) | 4.0(4.5) | 5.0(6.5) |

**Supplementary Table 4 | Symptoms and comorbidities reported by tested positive responders from top 5 countries**

|  | **SARS-COV-2 status** | | | |
| --- | --- | --- | --- | --- |
|  | **All Responders**  **n(%)** | **Tested**  **Positive**  **n(%)** | **Untested**  **Symptomatic**  **n(%)** | **Tested**  **Negative**  **n(%)** |
| **Mexico** | | | | |
| Answered questions on chronic conditions | 20616 | 2199 | 17432 | 985 |
| Reported one or more chronic conditions | 8256(40.0) | 913(41.5) | 6904(39.6) | 439(44.6) |
| Asthma | 763(3.7) | 87(4.0) | 633(3.6) | 43(4.4) |
| Diabetes type I | 260(1.3) | 32(1.5) | 213(1.2) | 15(1.5) |
| Diabetes type II | 968(4.7) | 132(6.0) | 778(4.5) | 58(5.9) |
| Heart disease | 181(0.9) | 12(0.5) | 149(0.9) | 20(2.0) |
| Hypertension | 2306(11.2) | 266(12.1) | 1895(10.9) | 145(14.7) |
| Kidney disease | 72(0.3) | 6(0.3) | 62(0.4) | 4(0.4) |
| Liver disease | 182(0.9) | 28(1.3) | 142(0.8) | 12(1.2) |
| Lung condition | 103(0.5) | 9(0.4) | 87(0.5) | 7(0.7) |
| Obesity | 5468(26.5) | 595(27.1) | 4620(26.5) | 253(25.7) |
| Answered questions on symptoms | 20616 | 2199 | 17432 | 985 |
| Chest pain | 444(2.2) | 76(3.5) | 343(2.0) | 25(2.5) |
| Chills | 4182(20.3) | 385(17.5) | 3593(20.6) | 204(20.7) |
| Cough | 8053(39.1) | 929(42.2) | 6757(38.8) | 367(37.3) |
| Diarrhoea | 4724(22.9) | 581(26.4) | 3920(22.5) | 223(22.6) |
| Fatigue | 10808(52.4) | 1270(57.8) | 9027(51.8) | 511(51.9) |
| Headache | 10877(52.8) | 1073(48.8) | 9311(53.4) | 493(50.1) |
| Itchy eyes | 3484(16.9) | 393(17.9) | 2917(16.7) | 174(17.7) |
| Joint pain | 413(2.0) | 59(2.7) | 326(1.9) | 28(2.8) |
| Loss of Appetite | 1664(8.1) | 272(12.4) | 1288(7.4) | 104(10.6) |
| Loss of smell and taste | 7786(37.8) | 1038(47.2) | 6434(36.9) | 314(31.9) |
| Muscle Ache | 8158(39.6) | 831(37.8) | 6957(39.9) | 370(37.6) |
| Nasal Congestion | 8113(39.4) | 714(32.5) | 7037(40.4) | 362(36.8) |
| Nausea and vomiting | 980(4.8) | 107(4.9) | 838(4.8) | 35(3.6) |
| Shortness of breath | 2601(12.6) | 328(14.9) | 2165(12.4) | 108(11.0) |
| Sore throat | 9055(43.9) | 721(32.8) | 7910(45.4) | 424(43.0) |
| Sputum | 1607(7.8) | 156(7.1) | 1376(7.9) | 75(7.6) |
| Temperature | 4774(23.2) | 441(20.1) | 4137(23.7) | 196(19.9) |
| **Brazil** |  |  |  |  |
| Answered questions on chronic conditions | 7257 | 1366 | 5089 | 802 |
| Reported one or more chronic conditions | 3026(41.7) | 556(40.7) | 2121(41.7) | 349(43.5) |
| Asthma | 425(5.9) | 74(5.4) | 302(5.9) | 49(6.1) |
| Diabetes type I | 54(0.7) | 13(1.0) | 30(0.6) | 11(1.4) |
| Diabetes type II | 262(3.6) | 56(4.1) | 170(3.3) | 36(4.5) |
| Heart disease | 120(1.7) | 24(1.8) | 76(1.5) | 20(2.5) |
| Hypertension | 1460(20.1) | 281(20.6) | 993(19.5) | 186(23.2) |
| Kidney disease | 41(0.6) | 5(0.4) | 28(0.6) | 8(1.0) |
| Liver disease | 200(2.8) | 23(1.7) | 162(3.2) | 15(1.9) |
| Lung condition | 65(0.9) | 3(0.2) | 58(1.1) | 4(0.5) |
| Obesity | 1395(19.2) | 248(18.2) | 982(19.3) | 165(20.6) |
| Answered questions on symptoms | 7257 | 1366 | 5089 | 802 |
| Chest pain | 1095(15.1) | 230(16.8) | 708(13.9) | 157(19.6) |
| Chills | 1587(21.9) | 255(18.7) | 1144(22.5) | 188(23.4) |
| Cough | 2856(39.4) | 532(38.9) | 2016(39.6) | 308(38.4) |
| Diarrhoea | 1548(21.3) | 327(23.9) | 1038(20.4) | 183(22.8) |
| Fatigue | 3096(42.7) | 664(48.6) | 2074(40.8) | 358(44.6) |
| Headache | 3889(53.6) | 691(50.6) | 2748(54.0) | 450(56.1) |
| Itchy eyes | 1558(21.5) | 259(19.0) | 1109(21.8) | 190(23.7) |
| Joint pain | 1491(20.6) | 261(19.1) | 1052(20.7) | 178(22.2) |
| Loss of Appetite | 1461(20.1) | 331(24.2) | 953(18.7) | 177(22.1) |
| Loss of smell and taste | 2453(33.8) | 695(50.9) | 1510(29.7) | 248(30.9) |
| Muscle Ache | 2315(31.9) | 401(29.4) | 1648(32.4) | 266(33.2) |
| Nasal Congestion | 3203(44.1) | 458(33.5) | 2390(47.0) | 355(44.3) |
| Nausea and vomiting | 300(4.1) | 65(4.8) | 199(3.9) | 36(4.5) |
| Shortness of breath | 582(8.0) | 114(8.3) | 385(7.6) | 83(10.3) |
| Sore throat | 2557(35.2) | 313(22.9) | 1961(38.5) | 283(35.3) |
| Sputum | 895(12.3) | 135(9.9) | 671(13.2) | 89(11.1) |
| Temperature | 965(13.3) | 122(8.9) | 740(14.5) | 103(12.8) |
| **Pakistan** |  |  |  |  |
| Answered questions on chronic conditions | 7451 | 753 | 6286 | 412 |
| Reported one or more chronic conditions | 2594(34.8) | 302(40.1) | 2115(33.6) | 177(43.0) |
| Asthma | 342(4.6) | 36(4.8) | 284(4.5) | 22(5.3) |
| Diabetes type I | 128(1.7) | 27(3.6) | 95(1.5) | 6(1.5) |
| Diabetes type II | 355(4.8) | 48(6.4) | 284(4.5) | 23(5.6) |
| Heart disease | 93(1.2) | 15(2.0) | 72(1.1) | 6(1.5) |
| Hypertension | 765(10.3) | 103(13.7) | 605(9.6) | 57(13.8) |
| Kidney disease | 66(0.9) | 11(1.5) | 51(0.8) | 4(1.0) |
| Liver disease | 194(2.6) | 29(3.9) | 151(2.4) | 14(3.4) |
| Lung condition | 82(1.1) | 8(1.1) | 69(1.1) | 5(1.2) |
| Obesity | 1184(15.9) | 117(15.5) | 993(15.8) | 74(18.0) |
| Answered questions on symptoms | 7451 | 753 | 6286 | 412 |
| Chest pain | 718(9.9) | 96(12.9) | 578(9.5) | 44(11.1) |
| Chills | 885(11.9) | 75(10.0) | 753(12.0) | 57(13.8) |
| Cough | 3259(43.7) | 308(40.9) | 2770(44.1) | 181(43.9) |
| Diarrhoea | 1086(14.6) | 172(22.8) | 864(13.7) | 50(12.1) |
| Fatigue | 4236(56.9) | 398(52.9) | 3596(57.2) | 242(58.7) |
| Headache | 2811(37.7) | 253(33.6) | 2398(38.1) | 160(38.8) |
| Itchy eyes | 793(11.0) | 65(8.7) | 683(11.2) | 45(11.4) |
| Joint pain | 1131(15.7) | 119(16.0) | 952(15.7) | 60(15.2) |
| Loss of Appetite | 826(11.4) | 125(16.8) | 649(10.7) | 52(13.2) |
| Loss of smell and taste | 2325(31.2) | 311(41.3) | 1907(30.3) | 107(26.0) |
| Muscle Ache | 2161(29.0) | 189(25.1) | 1850(29.4) | 122(29.6) |
| Nasal Congestion | 2305(30.9) | 207(27.5) | 1976(31.4) | 122(29.6) |
| Nausea and vomiting | 412(5.5) | 57(7.6) | 332(5.3) | 23(5.6) |
| Shortness of breath | 1028(13.8) | 114(15.1) | 859(13.7) | 55(13.3) |
| Sore throat | 3103(41.6) | 247(32.8) | 2679(42.6) | 177(43.0) |
| Sputum | 1430(19.2) | 118(15.7) | 1225(19.5) | 87(21.1) |
| Temperature | 2736(36.7) | 270(35.9) | 2327(37.0) | 139(33.7) |
| **India** |  |  |  |  |
| Answered questions on chronic conditions | 8967 | 714 | 7552 | 701 |
| Reported one or more chronic conditions | 2903(32.4) | 281(39.4) | 2331(30.9) | 291(41.5) |
| Asthma | 318(3.5) | 23(3.2) | 261(3.5) | 34(4.9) |
| Diabetes type I | 116(1.3) | 20(2.8) | 87(1.2) | 9(1.3) |
| Diabetes type II | 454(5.1) | 82(11.5) | 325(4.3) | 47(6.7) |
| Heart disease | 91(1.0) | 18(2.5) | 63(0.8) | 10(1.4) |
| Hypertension | 906(10.1) | 94(13.2) | 737(9.8) | 75(10.7) |
| Kidney disease | 86(1.0) | 6(0.8) | 66(0.9) | 14(2.0) |
| Liver disease | 179(2.0) | 32(4.5) | 122(1.6) | 25(3.6) |
| Lung condition | 70(0.8) | 13(1.8) | 47(0.6) | 10(1.4) |
| Obesity | 1316(14.7) | 99(13.9) | 1084(14.4) | 133(19.0) |
| Answered questions on symptoms | 8967 | 714 | 7552 | 701 |
| Chest pain | 494(6.3) | 60(8.8) | 383(5.9) | 51(7.9) |
| Chills | 1015(11.3) | 72(10.1) | 883(11.7) | 60(8.6) |
| Cough | 3433(38.3) | 266(37.3) | 2950(39.1) | 217(31.0) |
| Diarrhoea | 910(10.1) | 90(12.6) | 761(10.1) | 59(8.4) |
| Fatigue | 3577(39.9) | 280(39.2) | 3073(40.7) | 224(32.0) |
| Headache | 2567(28.6) | 163(22.8) | 2251(29.8) | 153(21.8) |
| Itchy eyes | 504(6.5) | 50(7.3) | 414(6.4) | 40(6.2) |
| Joint pain | 512(6.6) | 57(8.3) | 415(6.4) | 40(6.2) |
| Loss of Appetite | 525(6.7) | 83(12.2) | 397(6.1) | 45(7.0) |
| Loss of smell and taste | 1801(20.1) | 228(31.9) | 1478(19.6) | 95(13.6) |
| Muscle Ache | 2199(24.5) | 139(19.5) | 1909(25.3) | 151(21.5) |
| Nasal Congestion | 2413(26.9) | 151(21.1) | 2081(27.6) | 181(25.8) |
| Nausea and vomiting | 363(4.0) | 39(5.5) | 299(4.0) | 25(3.6) |
| Shortness of breath | 1401(15.6) | 62(8.7) | 1225(16.2) | 114(16.3) |
| Sore throat | 3669(40.9) | 181(25.4) | 3250(43.0) | 238(34.0) |
| Sputum | 1714(19.1) | 92(12.9) | 1506(19.9) | 116(16.5) |
| Temperature | 2017(22.5) | 206(28.9) | 1674(22.2) | 137(19.5) |
| **United Kingdom** |  |  |  |  |
| Answered questions on chronic conditions | 3055 | 427 | 2447 | 181 |
| Reported one or more chronic conditions | 1039(34.0) | 154(36.1) | 814(33.3) | 71(39.2) |
| Asthma | 358(11.7) | 54(12.6) | 278(11.4) | 26(14.4) |
| Diabetes type I | 18(0.6) | 3(0.7) | 14(0.6) | 1(0.6) |
| Diabetes type II | 127(4.2) | 19(4.4) | 100(4.1) | 8(4.4) |
| Heart disease | 50(1.6) | 4(0.9) | 41(1.7) | 5(2.8) |
| Hypertension | 385(12.6) | 61(14.3) | 302(12.3) | 22(12.2) |
| Kidney disease | 15(0.5) | 1(0.2) | 11(0.4) | 3(1.7) |
| Liver disease | 76(2.5) | 6(1.4) | 64(2.6) | 6(3.3) |
| Lung condition | 20(0.7) | 4(0.9) | 14(0.6) | 2(1.1) |
| Obesity | 389(12.7) | 61(14.3) | 299(12.2) | 29(16.0) |
| Answered questions on symptoms | 3055 | 427 | 2447 | 181 |
| Chest pain | 27(1.5) | 3(1.0) | 23(1.6) | 1(0.7) |
| Chills | 1191(39.0) | 161(37.7) | 964(39.4) | 66(36.5) |
| Cough | 1821(59.6) | 262(61.4) | 1446(59.1) | 113(62.4) |
| Diarrhoea | 812(26.6) | 102(23.9) | 661(27.0) | 49(27.1) |
| Fatigue | 2399(78.5) | 344(80.6) | 1918(78.4) | 137(75.7) |
| Headache | 1874(61.3) | 274(64.2) | 1491(60.9) | 109(60.2) |
| Itchy eyes | 44(2.4) | 7(2.4) | 33(2.4) | 4(2.6) |
| Joint pain | 43(2.3) | 7(2.4) | 35(2.5) | 1(0.7) |
| Loss of Appetite | 32(1.7) | 2(0.7) | 29(2.1) | 1(0.7) |
| Loss of smell and taste | 1013(33.2) | 248(58.1) | 715(29.2) | 50(27.6) |
| Muscle Ache | 1887(61.8) | 277(64.9) | 1498(61.2) | 112(61.9) |
| Nasal Congestion | 1203(39.4) | 171(40.0) | 969(39.6) | 63(34.8) |
| Nausea and vomiting | 313(10.2) | 39(9.1) | 249(10.2) | 25(13.8) |
| Shortness of breath | 1413(46.3) | 228(53.4) | 1104(45.1) | 81(44.8) |
| Sore throat | 1582(51.8) | 178(41.7) | 1318(53.9) | 86(47.5) |
| Sputum | 1215(39.8) | 160(37.5) | 992(40.5) | 63(34.8) |
| Temperature | 270(8.8) | 31(7.3) | 222(9.1) | 17(9.4) |
| **Peru** |  |  |  |  |
| Answered questions on chronic conditions | 3590 | 427 | 2618 | 545 |
| Reported one or more chronic conditions | 1506(41.9) | 166(38.9) | 1102(42.1) | 238(43.7) |
| Asthma | 229(6.4) | 22(5.2) | 165(6.3) | 42(7.7) |
| Diabetes type I | 20(0.6) | 3(0.7) | 17(0.6) | 0(0.0) |
| Diabetes type II | 137(3.8) | 10(2.3) | 97(3.7) | 30(5.5) |
| Heart disease | 48(1.3) | 6(1.4) | 35(1.3) | 7(1.3) |
| Hypertension | 370(10.3) | 52(12.2) | 255(9.7) | 63(11.6) |
| Kidney disease | 39(1.1) | 3(0.7) | 31(1.2) | 5(0.9) |
| Liver disease | 75(2.1) | 10(2.3) | 50(1.9) | 15(2.8) |
| Lung condition | 30(0.8) | 4(0.9) | 22(0.8) | 4(0.7) |
| Obesity | 860(24.0) | 92(21.5) | 650(24.8) | 118(21.7) |
| Answered questions on symptoms | 3590 | 427 | 2618 | 545 |
| Chest pain | 41(1.1) | 7(1.6) | 24(0.9) | 10(1.8) |
| Chills | 805(22.4) | 80(18.7) | 620(23.7) | 105(19.3) |
| Cough | 1469(40.9) | 146(34.2) | 1114(42.6) | 209(38.3) |
| Diarrhoea | 820(22.8) | 106(24.8) | 586(22.4) | 128(23.5) |
| Fatigue | 1474(41.1) | 149(34.9) | 1116(42.6) | 209(38.3) |
| Headache | 1735(48.3) | 161(37.7) | 1336(51.0) | 238(43.7) |
| Itchy eyes | 433(12.1) | 61(14.3) | 300(11.5) | 72(13.2) |
| Joint pain | 17(0.5) | 4(0.9) | 8(0.3) | 5(0.9) |
| Loss of Appetite | 115(3.2) | 17(4.0) | 71(2.7) | 27(5.0) |
| Loss of smell and taste | 1027(28.6) | 102(23.9) | 776(29.6) | 149(27.3) |
| Muscle Ache | 1344(37.4) | 132(30.9) | 1029(39.3) | 183(33.6) |
| Nasal Congestion | 1498(41.7) | 135(31.6) | 1164(44.5) | 199(36.5) |
| Nausea and vomiting | 141(3.9) | 12(2.8) | 108(4.1) | 21(3.9) |
| Shortness of breath | 588(16.4) | 53(12.4) | 470(18.0) | 65(11.9) |
| Sore throat | 1615(45.0) | 134(31.4) | 1255(47.9) | 226(41.5) |
| Sputum | 631(17.6) | 42(9.8) | 520(19.9) | 69(12.7) |
| Temperature | 578(16.1) | 51(11.9) | 431(16.5) | 96(17.6) |
| **Bolivia** |  |  |  |  |
| Answered questions on chronic conditions | 4385 | 388 | 3639 | 358 |
| Reported one or more chronic conditions | 1472(33.6) | 136(35.1) | 1204(33.1) | 132(36.9) |
| Asthma | 110(2.5) | 10(2.6) | 91(2.5) | 9(2.5) |
| Diabetes type I | 24(0.5) | 0(0.0) | 21(0.6) | 3(0.8) |
| Diabetes type II | 101(2.3) | 13(3.4) | 78(2.1) | 10(2.8) |
| Heart disease | 47(1.1) | 8(2.1) | 35(1.0) | 4(1.1) |
| Hypertension | 397(9.1) | 46(11.9) | 317(8.7) | 34(9.5) |
| Kidney disease | 45(1.0) | 3(0.8) | 33(0.9) | 9(2.5) |
| Liver disease | 68(1.6) | 5(1.3) | 58(1.6) | 5(1.4) |
| Lung condition | 29(0.7) | 5(1.3) | 23(0.6) | 1(0.3) |
| Obesity | 900(20.5) | 70(18.0) | 743(20.4) | 87(24.3) |
| Answered questions on symptoms | 4385 | 388 | 3639 | 358 |
| Chest pain | 101(2.3) | 12(3.1) | 76(2.1) | 13(3.6) |
| Chills | 949(21.6) | 56(14.4) | 834(22.9) | 59(16.5) |
| Cough | 1638(37.4) | 120(30.9) | 1406(38.6) | 112(31.3) |
| Diarrhoea | 954(21.8) | 88(22.7) | 789(21.7) | 77(21.5) |
| Fatigue | 1867(42.6) | 178(45.9) | 1556(42.8) | 133(37.2) |
| Headache | 2378(54.2) | 181(46.6) | 2013(55.3) | 184(51.4) |
| Itchy eyes | 1038(23.7) | 88(22.7) | 852(23.4) | 98(27.4) |
| Joint pain | 53(1.2) | 5(1.3) | 42(1.2) | 6(1.7) |
| Loss of Appetite | 277(6.3) | 26(6.7) | 227(6.2) | 24(6.7) |
| Loss of smell and taste | 1383(31.5) | 127(32.7) | 1174(32.3) | 82(22.9) |
| Muscle Ache | 1533(35.0) | 121(31.2) | 1296(35.6) | 116(32.4) |
| Nasal Congestion | 1990(45.4) | 131(33.8) | 1712(47.0) | 147(41.1) |
| Nausea and vomiting | 232(5.3) | 23(5.9) | 194(5.3) | 15(4.2) |
| Shortness of breath | 570(13.0) | 58(14.9) | 464(12.8) | 48(13.4) |
| Sore throat | 1934(44.1) | 127(32.7) | 1662(45.7) | 145(40.5) |
| Sputum | 533(12.2) | 31(8.0) | 467(12.8) | 35(9.8) |
| Temperature | 997(22.7) | 49(12.6) | 880(24.2) | 68(19.0) |
| **Chile** |  |  |  |  |
| Answered questions on chronic conditions | 1675 | 343 | 1202 | 130 |
| Reported one or more chronic conditions | 731(43.6) | 152(44.3) | 524(43.6) | 55(42.3) |
| Asthma | 115(6.9) | 21(6.1) | 88(7.3) | 6(4.6) |
| Diabetes type I | 6(0.4) | 1(0.3) | 3(0.2) | 2(1.5) |
| Diabetes type II | 80(4.8) | 20(5.8) | 55(4.6) | 5(3.8) |
| Heart disease | 25(1.5) | 3(0.9) | 19(1.6) | 3(2.3) |
| Hypertension | 226(13.5) | 51(14.9) | 159(13.2) | 16(12.3) |
| Kidney disease | 7(0.4) | 2(0.6) | 4(0.3) | 1(0.8) |
| Liver disease | 17(1.0) | 1(0.3) | 13(1.1) | 3(2.3) |
| Lung condition | 7(0.4) | 4(1.2) | 1(0.1) | 2(1.5) |
| Obesity | 454(27.1) | 93(27.1) | 329(27.4) | 32(24.6) |
| Answered questions on symptoms | 1675 | 343 | 1202 | 130 |
| Chest pain | 19(1.1) | 4(1.2) | 13(1.1) | 2(1.6) |
| Chills | 473(28.2) | 88(25.7) | 350(29.1) | 35(26.9) |
| Cough | 748(44.7) | 171(49.9) | 521(43.3) | 56(43.1) |
| Diarrhoea | 439(26.2) | 111(32.4) | 286(23.8) | 42(32.3) |
| Fatigue | 833(49.7) | 179(52.2) | 586(48.8) | 68(52.3) |
| Headache | 1092(65.2) | 208(60.6) | 808(67.2) | 76(58.5) |
| Itchy eyes | 244(14.6) | 62(18.1) | 164(13.6) | 18(14.0) |
| Joint pain | 10(0.6) | 5(1.5) | 5(0.4) | 0(0.0) |
| Loss of Appetite | 91(5.4) | 25(7.3) | 60(5.0) | 6(4.7) |
| Loss of smell and taste | 592(35.3) | 195(56.9) | 363(30.2) | 34(26.2) |
| Muscle Ache | 808(48.2) | 172(50.1) | 581(48.3) | 55(42.3) |
| Nasal Congestion | 801(47.8) | 181(52.8) | 560(46.6) | 60(46.2) |
| Nausea and vomiting | 105(6.3) | 33(9.6) | 63(5.2) | 9(6.9) |
| Shortness of breath | 302(18.0) | 74(21.6) | 202(16.8) | 26(20.0) |
| Sore throat | 708(42.3) | 105(30.6) | 544(45.3) | 59(45.4) |
| Sputum | 242(14.4) | 53(15.5) | 167(13.9) | 22(16.9) |
| Temperature | 244(14.6) | 60(17.5) | 169(14.1) | 15(11.5) |
| **Ecuador** |  |  |  |  |
| Answered questions on chronic conditions | 2163 | 282 | 1596 | 285 |
| Reported one or more chronic conditions | 655(30.3) | 85(30.1) | 481(30.1) | 89(31.2) |
| Asthma | 62(2.9) | 6(2.1) | 45(2.8) | 11(3.9) |
| Diabetes type I | 18(0.8) | 5(1.8) | 11(0.7) | 2(0.7) |
| Diabetes type II | 36(1.7) | 4(1.4) | 28(1.8) | 4(1.4) |
| Heart disease | 23(1.1) | 3(1.1) | 14(0.9) | 6(2.1) |
| Hypertension | 171(7.9) | 24(8.5) | 122(7.6) | 25(8.8) |
| Kidney disease | 12(0.6) | 0(0.0) | 8(0.5) | 4(1.4) |
| Liver disease | 35(1.6) | 4(1.4) | 26(1.6) | 5(1.8) |
| Lung condition | 9(0.4) | 2(0.7) | 6(0.4) | 1(0.4) |
| Obesity | 390(18.0) | 54(19.1) | 288(18.0) | 48(16.8) |
| Answered questions on symptoms | 2163 | 282 | 1596 | 285 |
| Chest pain | 29(1.3) | 7(2.5) | 15(0.9) | 7(2.5) |
| Chills | 480(22.2) | 51(18.1) | 367(23.0) | 62(21.8) |
| Cough | 838(38.7) | 104(36.9) | 639(40.0) | 95(33.3) |
| Diarrhoea | 539(24.9) | 87(30.9) | 380(23.8) | 72(25.3) |
| Fatigue | 935(43.2) | 131(46.5) | 687(43.0) | 117(41.1) |
| Headache | 1099(50.8) | 121(42.9) | 839(52.6) | 139(48.8) |
| Itchy eyes | 376(17.4) | 56(19.9) | 270(16.9) | 50(17.5) |
| Joint pain | 24(1.1) | 5(1.8) | 15(0.9) | 4(1.4) |
| Loss of Appetite | 107(5.0) | 23(8.2) | 73(4.6) | 11(3.9) |
| Loss of smell and taste | 663(30.7) | 114(40.4) | 473(29.6) | 76(26.7) |
| Muscle Ache | 799(36.9) | 102(36.2) | 594(37.2) | 103(36.1) |
| Nasal Congestion | 959(44.3) | 95(33.7) | 744(46.6) | 120(42.1) |
| Nausea and vomiting | 102(4.7) | 11(3.9) | 78(4.9) | 13(4.6) |
| Shortness of breath | 295(13.6) | 34(12.1) | 220(13.8) | 41(14.4) |
| Sore throat | 927(42.9) | 98(34.8) | 722(45.2) | 107(37.5) |
| Sputum | 285(13.2) | 29(10.3) | 231(14.5) | 25(8.8) |
| Temperature | 418(19.3) | 50(17.7) | 318(19.9) | 50(17.5) |
| **Guatemala** |  |  |  |  |
| Answered questions on chronic conditions | 1217 | 170 | 985 | 62 |
| Reported one or more chronic conditions | 453(37.2) | 68(40.0) | 364(37.0) | 21(33.9) |
| Asthma | 50(4.1) | 5(2.9) | 43(4.4) | 2(3.2) |
| Diabetes type I | 10(0.8) | 2(1.2) | 7(0.7) | 1(1.6) |
| Diabetes type II | 54(4.4) | 11(6.5) | 38(3.9) | 5(8.1) |
| Heart disease | 13(1.1) | 2(1.2) | 11(1.1) | 0(0.0) |
| Hypertension | 132(10.8) | 25(14.7) | 100(10.2) | 7(11.3) |
| Kidney disease | 12(1.0) | 2(1.2) | 10(1.0) | 0(0.0) |
| Liver disease | 13(1.1) | 2(1.2) | 10(1.0) | 1(1.6) |
| Lung condition | 4(0.3) | 0(0.0) | 4(0.4) | 0(0.0) |
| Obesity | 246(20.2) | 32(18.8) | 205(20.8) | 9(14.5) |
| Answered questions on symptoms | 1217 | 170 | 985 | 62 |
| Chest pain | 35(2.9) | 4(2.4) | 27(2.7) | 4(6.5) |
| Chills | 226(18.6) | 29(17.1) | 185(18.8) | 12(19.4) |
| Cough | 455(37.4) | 75(44.1) | 361(36.6) | 19(30.6) |
| Diarrhoea | 293(24.1) | 62(36.5) | 216(21.9) | 15(24.2) |
| Fatigue | 487(40.0) | 88(51.8) | 383(38.9) | 16(25.8) |
| Headache | 606(49.8) | 88(51.8) | 491(49.8) | 27(43.5) |
| Itchy eyes | 274(22.5) | 28(16.5) | 229(23.2) | 17(27.4) |
| Joint pain | 25(2.1) | 5(2.9) | 18(1.8) | 2(3.2) |
| Loss of Appetite | 97(8.0) | 23(13.5) | 69(7.0) | 5(8.1) |
| Loss of smell and taste | 428(35.2) | 93(54.7) | 323(32.8) | 12(19.4) |
| Muscle Ache | 399(32.8) | 53(31.2) | 327(33.2) | 19(30.6) |
| Nasal Congestion | 502(41.2) | 74(43.5) | 402(40.8) | 26(41.9) |
| Nausea and vomiting | 52(4.3) | 8(4.7) | 39(4.0) | 5(8.1) |
| Shortness of breath | 129(10.6) | 16(9.4) | 107(10.9) | 6(9.7) |
| Sore throat | 468(38.5) | 43(25.3) | 401(40.7) | 24(38.7) |
| Sputum | 72(5.9) | 12(7.1) | 58(5.9) | 2(3.2) |
| Temperature | 317(26.0) | 37(21.8) | 268(27.2) | 12(19.4) |
| **Honduras** |  |  |  |  |
| Answered questions on chronic conditions | 1168 | 165 | 808 | 195 |
| Reported one or more chronic conditions | 444(38.0) | 54(32.7) | 311(38.5) | 79(40.5) |
| Asthma | 84(7.2) | 15(9.1) | 52(6.4) | 17(8.7) |
| Diabetes type I | 13(1.1) | 2(1.2) | 7(0.9) | 4(2.1) |
| Diabetes type II | 33(2.8) | 6(3.6) | 25(3.1) | 2(1.0) |
| Heart disease | 3(0.3) | 0(0.0) | 1(0.1) | 2(1.0) |
| Hypertension | 146(12.5) | 17(10.3) | 104(12.9) | 25(12.8) |
| Kidney disease | 2(0.2) | 0(0.0) | 1(0.1) | 1(0.5) |
| Liver disease | 15(1.3) | 2(1.2) | 9(1.1) | 4(2.1) |
| Lung condition | 6(0.5) | 0(0.0) | 4(0.5) | 2(1.0) |
| Obesity | 237(20.3) | 21(12.7) | 180(22.3) | 36(18.5) |
| Answered questions on symptoms | 1168 | 165 | 808 | 195 |
| Chest pain | 34(2.9) | 14(8.5) | 15(1.9) | 5(2.6) |
| Chills | 167(14.3) | 27(16.4) | 111(13.7) | 29(14.9) |
| Cough | 387(33.1) | 49(29.7) | 278(34.4) | 60(30.8) |
| Diarrhoea | 231(19.8) | 42(25.5) | 148(18.3) | 41(21.0) |
| Fatigue | 468(40.1) | 65(39.4) | 316(39.1) | 87(44.6) |
| Headache | 584(50.0) | 80(48.5) | 400(49.5) | 104(53.3) |
| Itchy eyes | 236(20.2) | 41(24.8) | 147(18.2) | 48(24.6) |
| Joint pain | 28(2.4) | 4(2.4) | 13(1.6) | 11(5.6) |
| Loss of Appetite | 89(7.6) | 16(9.7) | 52(6.5) | 21(10.8) |
| Loss of smell and taste | 392(33.6) | 62(37.6) | 261(32.3) | 69(35.4) |
| Muscle Ache | 384(32.9) | 44(26.7) | 264(32.7) | 76(39.0) |
| Nasal Congestion | 505(43.2) | 51(30.9) | 365(45.2) | 89(45.6) |
| Nausea and vomiting | 51(4.4) | 9(5.5) | 31(3.8) | 11(5.6) |
| Shortness of breath | 113(9.7) | 19(11.5) | 74(9.2) | 20(10.3) |
| Sore throat | 451(38.6) | 48(29.1) | 328(40.6) | 75(38.5) |
| Sputum | 85(7.3) | 8(4.8) | 66(8.2) | 11(5.6) |
| Temperature | 272(23.3) | 39(23.6) | 182(22.5) | 51(26.2) |
| **Dominican Republic** |  |  |  |  |
| Answered questions on chronic conditions | 1244 | 145 | 956 | 143 |
| Reported one or more chronic conditions | 481(38.7) | 46(31.7) | 374(39.1) | 61(42.7) |
| Asthma | 100(8.0) | 8(5.5) | 77(8.1) | 15(10.5) |
| Diabetes type I | 15(1.2) | 3(2.1) | 10(1.0) | 2(1.4) |
| Diabetes type II | 41(3.3) | 8(5.5) | 25(2.6) | 8(5.6) |
| Heart disease | 13(1.0) | 1(0.7) | 10(1.0) | 2(1.4) |
| Hypertension | 199(16.0) | 20(13.8) | 153(16.0) | 26(18.2) |
| Kidney disease | 3(0.2) | 0(0.0) | 3(0.3) | 0(0.0) |
| Liver disease | 13(1.0) | 1(0.7) | 10(1.0) | 2(1.4) |
| Lung condition | 12(1.0) | 2(1.4) | 9(0.9) | 1(0.7) |
| Obesity | 227(18.2) | 22(15.2) | 178(18.6) | 27(18.9) |
| Answered questions on symptoms | 1244 | 145 | 956 | 143 |
| Chest pain | 33(2.7) | 6(4.1) | 24(2.5) | 3(2.1) |
| Chills | 274(22.0) | 28(19.3) | 219(22.9) | 27(18.9) |
| Cough | 444(35.7) | 57(39.3) | 329(34.4) | 58(40.6) |
| Diarrhoea | 270(21.7) | 43(29.7) | 202(21.1) | 25(17.5) |
| Fatigue | 609(49.0) | 66(45.5) | 472(49.4) | 71(49.7) |
| Headache | 684(55.0) | 62(42.8) | 546(57.1) | 76(53.1) |
| Itchy eyes | 268(21.6) | 31(21.4) | 210(22.0) | 27(18.9) |
| Joint pain | 25(2.0) | 3(2.1) | 18(1.9) | 4(2.8) |
| Loss of Appetite | 145(11.7) | 30(20.7) | 96(10.1) | 19(13.3) |
| Loss of smell and taste | 522(42.0) | 83(57.2) | 379(39.6) | 60(42.0) |
| Muscle Ache | 566(45.5) | 55(37.9) | 452(47.3) | 59(41.3) |
| Nasal Congestion | 532(42.8) | 49(33.8) | 420(43.9) | 63(44.1) |
| Nausea and vomiting | 47(3.8) | 6(4.1) | 39(4.1) | 2(1.4) |
| Shortness of breath | 165(13.3) | 20(13.8) | 128(13.4) | 17(11.9) |
| Sore throat | 419(33.7) | 31(21.4) | 336(35.1) | 52(36.4) |
| Sputum | 87(7.0) | 8(5.5) | 63(6.6) | 16(11.2) |
| Temperature | 360(28.9) | 41(28.3) | 276(28.9) | 43(30.1) |

**Supplementary Table 5 | Symptoms reported by tested positive respondents with underlying chronic preconditions.**

|  | **No conditions**  **n(%)** | **Asthma n(%)** | **Lung condition n(%)** | **Type 1 diabetes n(%)** | **Type 2 diabetes n(%)** | **Hypertension n(%)** | **Obesity n(%)** | **Heart disease n(%)** | **Liver disease n(%)** | **Kidney disease n(%)** |
| --- | --- | --- | --- | --- | --- | --- | --- | --- | --- | --- |
| Total count | 4813 | 265 | 99 | 47 | 203 | 593 | 1153 | 44 | 23 | 20 |
| Chest pain | 316  (6.8) | 32 (12.4) | 14 (15.2) | 3 (6.4) | 9 (4.6) | 37 (6.5) | 67 (5.9) | 4 (9.3) | 2 (8.7) | 2 (10.0) |
| Chills | 821 (17.1) | 71 (26.8) | 13 (13.1) | 4 (8.5) | 33 (16.3) | 116 (19.6) | 185 (16.0) | 11 (25.0) | 4 (17.4) | 7 (35.0) |
| Cough | 1853 (38.5) | 117 (44.2) | 48 (48.5) | 23 (48.9) | 86 (42.4) | 243 (41.0) | 511 (44.3) | 24 (54.5) | 13 (56.5) | 10 (50.0) |
| Diarrhoea | 1094 (22.7) | 83 (31.3) | 21 (21.2) | 10 (21.3) | 47 (23.2) | 150 (25.3) | 310 (26.9) | 16 (36.4) | 2 (8.7) | 5 (25.0) |
| Fatigue | 2395 (49.8) | 165 (62.3) | 43 (43.4) | 17 (36.2) | 86 (42.4) | 319 (53.8) | 614 (53.3) | 26 (59.1) | 12 (52.2) | 11 (55.0) |
| Headache | 2202 (45.8) | 149 (56.2) | 32 (32.3) | 12 (25.5) | 73 (36.0) | 264 (44.5) | 534 (46.3) | 19 (43.2) | 7 (30.4) | 4 (20.0) |
| Itchy eyes | 719 (15.4) | 45 (17.4) | 7 (7.6) | 4 (8.5) | 28 (14.4) | 81 (14.3) | 184 (16.1) | 10 (23.3) | 2 (8.7) | 1 (5.0) |
| Joint pain | 330 (7.1) | 30 (11.6) | 6 (6.5) | 6 (12.8) | 13 (6.7) | 47 (8.3) | 58 (5.1) | 4 (9.3) | 0 (0.0) | 3 (15.0) |
| Loss of Appetite | 619 (13.3) | 44 (17.0) | 9 (9.8) | 6 (12.8) | 26 (13.4) | 70 (12.3) | 143 (12.5) | 8 (18.6) | 2 (8.7) | 2 (10.0) |
| Loss of smell and taste | 2214 (46.0) | 106 (40.0) | 29 (29.3) | 10 (21.3) | 68 (33.5) | 224 (37.8) | 558 (48.4) | 19 (43.2) | 11 (47.8) | 7 (35.0) |
| Muscle Ache | 1601 (33.3) | 101 (38.1) | 28 (28.3) | 8 (17.0) | 59 (29.1) | 219 (36.9) | 392 (34.0) | 19 (43.2) | 8 (34.8) | 11 (55.0) |
| Nasal Congestion | 1634 (33.9) | 93 (35.1) | 22 (22.2) | 3 (6.4) | 47 (23.2) | 159 (26.8) | 400 (34.7) | 17 (38.6) | 5 (21.7) | 5 (25.0) |
| Nausea and vomiting | 248 (5.2) | 17 (6.4) | 8 (8.1) | 1 (2.1) | 16 (7.9) | 25 (4.2) | 65 (5.6) | 5 (11.4) | 0 (0.0) | 2 (10.0) |
| Shortness of breath | 657 (13.7) | 67 (25.3) | 24 (24.2) | 5 (10.6) | 22 (10.8) | 104 (17.5) | 200 (17.3) | 10 (22.7) | 5 (21.7) | 1 (5.0) |
| Sore throat | 1474 (30.6) | 93 (35.1) | 30 (30.3) | 14 (29.8) | 51 (25.1) | 161 (27.2) | 327 (28.4) | 18 (40.9) | 9 (39.1) | 9 (45.0) |
| Sputum | 517 (10.7) | 46 (17.4) | 22 (22.2) | 11 (23.4) | 17 (8.4) | 62 (10.5) | 131 (11.4) | 6 (13.6) | 2 (8.7) | 2 (10.0) |
| Temperature | 847 (17.6) | 45 (17.0) | 22 (22.2) | 15 (31.9) | 53 (26.1) | 121 (20.4) | 208 (18.0) | 10 (22.7) | 6 (26.1) | 3 (15.0) |

**Supplementary Table 6 | Model tables of the logistic regression models presented in Figure 5. The table is also added as an additional file along with the manuscript**

**Supplementary Table 7 | Comparison of most reported symptoms (normally >33%) in our global survey and major and minor symptoms as stated by selected national and international public health agencies**

| **Country** | **Common symptoms (reported by >33%) in our global survey** | **Official main/most common symptoms** | **Official additional/Less common symptoms** | **Ref** |
| --- | --- | --- | --- | --- |
| Mexico | Fatigue  Headache  Loss of smell and taste  Cough  Muscle ache | At least two of the following:  Cough/sneezing,  Fever,  Headache | Breathing difficulty  Throat pain  Runny nose  Red eyes  Muscle/joint pain | ^1^ |
| Brazil | Headache  Nasal congestion  Fatigue  Cough  Sore throat  Loss of smell and taste | Cough  Sore throat  Runny nose  Fever | Anosmia  Ageusia  Diarrhea  Abdominal pain  Chills  Myalgia  Fatigue Headache  Adynamia  Prostration  Hyperoxia  Diarrhea | ^2^ |
| Pakistan | Fatigue  Loss of smell and taste  Cough  Headache  Temperature | Respiratory symptoms (cough and shortness of breath),  Fever ,  Muscle pain,  Headache,  Sore throat,  New loss of taste or smell,  Severe fatigue |  | ^3^ |
| India | Fatigue  Cough | Fever  Tiredness  Cough | Aches and pains  Nasal congestion  Runny nose  Sore throat  Diarrhoea | ^4^ |
| UK | Fatigue  Muscle ache  Headache  Cough  Loss of smell and taste  Shortness of breath  Sore throat  Nasal congestion  Chills  Sputum | New persistent cough High temperature Loss of taste and smell. |  | ^5^ |
| Peru | Headache  Fatigue  Cough | Fever  Dry cough  Tiredness  Throat pain  Difficulty breathing  Nasal congestion |  | ^6^ |
| Bolivia | Headache  Fatigue  Nasal congestion | Fever  Dry cough  Tiredness | Loss of taste or smell  Nasal congestion  Conjunctivitis (eye redness)  Throat pain  Headache  Muscle or joint pain  Different types of skin rashes  Nausea or vomiting  Diarrhea  Chills or vertigo | ^7^ |
| Chile | Headache  Loss of smell and taste  Nasal congestion  Fatigue  Muscle ache  Cough | Fever  Cough  Dyspnea or shortness of breath  Odynophagia  Headache | Myalgia or muscle aches.  Chills  Diarrhea.  Sudden loss of smell  Sudden loss of taste | ^8^ |
| Ecuador | Fatigue  Headache  Loss of smell and taste  Cough  Muscle ache  Sore throat  Nasal congestion | Fever or chills  Cough  Tiredness  Shortness of breath (shortness of breath) | Muscle and body aches  Headache  Recent loss of smell or taste  Sore throat  Congestion or runny nose  Nausea or vomiting  Diarrhea  Conjunctivitis  Rashes or color changes in the fingers or toes. | ^9^ |
| Guatemala | Loss of smell and taste  Fatigue  Headache  Cough  Nasal congestion  Diarrhoea | Fever  Headache  Coughing/sneezing  Difficulty breathing  General discomfort |  | ^10^ |
| Honduras | Headache  Fatigue  Loss of smell and taste | Fever  Dry cough  Difficulty breathing  Headache  Throat pain |  | ^11^ |
| Dominican Republic | Loss of smell and taste  Fatigue  Headache  Cough  Muscle ache  Nasal congestion | Fever  Tiredness  Dry cough | Pain  Nasal congestion  Runny nose  Sore throat  Diarrhea | ^12^ |
| WHO | [reporting global statistics as proxy]  Fatigue  Headache  Loss of smell and taste  Cough  Muscle ache  Nasal congestion | Fever  Tiredness  Dry cough | Loss of taste or smell  Nasal congestion  Conjunctivitis (also known as red eyes)  Sore throat  Headache  Muscle or joint pain  Different types of skin rash  Nausea or vomiting  Diarrhea  Chills or dizziness | ^13^ |

**Supplementary References:**

1. Government of Mexico. <https://coronavirus.gob.mx/covid-19/> (accessed on June 01, 2021).

2. Ministry of Health, Brazil. <https://www.gov.br/saude/pt-br/coronavirus/sintomas> (accessed on June 01, 2021).

3. Ministry of National Healt Services, Government of Pakistan. <https://storage.covid.gov.pk/new_guidelines/11December2020_20201211_Clinical_Management_Guidelines_for_COVID-19_infection_1204.pdf> (accessed on June 01, 2021).

4. Ministry of Health and Family Welfare, Government of India. <https://www.mohfw.gov.in/pdf/FAQ.pdf> (accessed on June 01, 2021).

5. NHS UK. <https://www.nhs.uk/conditions/coronavirus-covid-19/symptoms/> (accessed on June 01, 2021).

6. Government of Peru. <https://www.gob.pe/8371-que-son-los-coronavirus/pages> (accessed on June 01, 2021).

7. Estado Plurinacional de Bolivia <https://www.boliviasegura.gob.bo/index.php/que-es-el-covid/> accessed on June 01, 2021).

8. Government of Chile. <https://www.gob.cl/coronavirus/> (accessed on June 01, 2021).

9. Government of Ecuador. <https://www.coronavirusecuador.com/faq/> (accessed on June 01, 2021).

10. Government of Guatemala. <https://covid19.gob.gt/> (accessed on June 01, 2021).

11. Government of Honduras. <https://covid19honduras.org/> (accessed on June 01, 2021).

12. Government of Dominican Republic. <https://coronavirusrd.gob.do/wp-content/uploads/2020/03/35-Preguntas-y-Respuestas-Sobre-el-Coronavirus-y-Falsos-Rumores.pdf.pdf.pdf> (accessed on June 01, 2021).

13. WHO. <https://www.who.int/emergencies/diseases/novel-coronavirus-2019/question-and-answers-hub/q-a-detail/coronavirus-disease-covid-19> (accessed on June 01, 2021).
